# The responses of lungs and adjacent lymph nodes in responding to *Yersinia pestis* infection: A transcriptomic study using a non-human primate model

**DOI:** 10.1371/journal.pone.0209592

**Published:** 2019-02-21

**Authors:** Nabarun Chakraborty, Aarti Gautam, Seid Muhie, Stacy-Ann Miller, Candace Moyler, Marti Jett, Rasha Hammamieh

**Affiliations:** 1 The Geneva Foundation, US Army Center for Environmental Health Research, Fort Detrick, MD, United States of America; 2 US Army Center for Environmental Health Research, Fort Detrick, MD, United States of America; 3 ORISE, US Army Center for Environmental Health Research, Fort Detrick, MD, United States of America; University of the Pacific, UNITED STATES

## Abstract

Initiation of treatment during the pre-symptomatic phase of *Yersinia pestis* (*Y*. *pestis*) infection is particularly critical. The rapid proliferation of *Y*. *pestis* typically couples with the manifestation of common flu-like early symptoms that often misguides the medical intervention. Our study used African green monkeys (AGM) that did not exhibit clear clinical symptoms for nearly two days after intranasal challenge with *Y*. *pestis* and succumbed within a day after showing the first signs of clinical symptoms. The lung, and mediastinal and submandibular lymph nodes (LN) accumulated significant *Y*. *pestis* colonization immediately after the intranasal challenge. Hence, organ-specific molecular investigations are deemed to be the key to elucidating mechanisms of the initial host response. Our previous study focused on the whole blood of AGM, and we found early perturbations in the ubiquitin-microtubule-mediated host defense. Altered expression of the genes present in ubiquitin and microtubule networks indicated an early suppression of these networks in the submandibular lymph nodes. In concert, the upstream toll-like receptor signaling and downstream NFκB signaling were inhibited at the multi-omics level. The inflammatory response was suppressed in the lungs, submandibular lymph nodes and mediastinal lymph nodes. We posited a causal chain of molecular mechanisms that indicated *Y*. *pestis* was probably able to impair host-mediated proteolysis activities and evade autophagosome capture by dysregulating both ubiquitin and microtubule networks in submandibular lymph nodes. Targeting these networks in a submandibular LN-specific and time-resolved fashion could be essential for development of the next generation therapeutics for pneumonic plague.

## Introduction

Time-sensitive diagnosis is the foremost challenge in the management of infection with *Yersinia pestis* (*Y*. *pestis*) [1, 2], the causative agent of pneumonic plague [3]. Rapid proliferation of *Y*. *pestis* during the pre-symptomatic phase allows very little time for therapeutic intervention. Therefore, identification of early markers of disease pathogenesis and of novel targets to inhibit the invasion is the fundamental objective of next generation therapeutic research.

Previously, we reported a longitudinal study in which African green monkeys (AGMs) were intranasally challenged with *Y*. *pestis* strain CO92 [4]. Our *in vivo* model was built on a past communication [5] that established AGM as the reliable model for plague experiments. We collected AGMs’ organs and blood at six different time-points post-infection (p.i.). The AGMs succumbed to death within 78 h p.i.; no obvious symptoms of disease including fever were detected until 48 h p.i., leaving essentially little more than one day to treat a moribund animal. Bacterial colonization in the blood was confirmed above the threshold level at nearly 32 h p.i., but transcriptomic investigation of blood samples indicated much earlier signs of pathogenesis [4], which compelled the investigation of the molecular landscape in greater detail.

*Blood transcriptomic assays suggested an early involvement of ubiquitination and microtubule activities in response to Y*. *pestis* [4]. The ubiquitin network controls a broad range of immunological activities including pathogen detection, antigen presentation, and proteolysis via proteasome-, phagolysosome- and autophagosome-mediated degradation [6]. In addition, ubiquitination of NFκB networks under stressed conditions essentially regulates a range of genes encoding cytokines and pro-inflammatory molecules [7]. Evidently the ubiquitination-deubiquitination process is a key battleground of host-pathogen interactions, as the pathogens typically attempt to manipulate the ubiquitination-deubiquitination mechanism in their favor [8, 9]. In light of past reports suggesting the potential effects of *Y*. *pestis* surface proteins on many genes enriching the NFκB network [10–12], the present study offered a unique opportunity to interrogate the longitudinal regulation of this network and its upstream and downstream regulators. The role of microtubules in *Y*. *pestis* uptake was suggested earlier [13, 14]. Microtubules further the sequestration of those proteins that escape the impaired proteolysis [15]. Hence, the manipulation of the microtubule architecture creates a replication-permissive niche that promotes the intracellular movement of the pathogen [16]. Clearly, studying these ubiquitin-microtubule mechanisms together can potentially enrich our understanding about *Y*. *pestis* pathogenesis.

Increasing numbers of reports have demonstrated how a gene cluster is preferentially perturbed in one organ type over other organ types under similar stress [17]. Therefore, we decided that an investigation of organ-specific molecular events was needed in order to interpret the cross-talk among various organs [18] in a biologically meaningful way. Post infection, the lungs, submandibular lymph nodes (LN) and mediastinal LN, were the major organs that showed the first signs of bacterial accumulation [4], sometimes a day before the colonization could be detected in blood. Therefore, these organs are likely to be the primary battlegrounds for host-pathogen interaction at the onset of *Y*. *pestis* challenge. A number of studies using various animal models supported the critical role of lungs and adjacent lymph nodes in the response to *Yersinia* infection [19–22]. Longitudinal transcriptomic analyses of these organs were primarily focused on the ubiquitin-microtubule networks and neighboring pathways.

Our organ-specific investigation showed the submandibular LN to be the most active organ during the early episode of pathogenesis. Multi-omics assays predicted a comprehensive inhibition of ubiquitin and microtubule networks, which was synchronized with the early onset of apoptosis and immunosuppression. Thereby a niche was potentially created to facilitate the rapid proliferation of *Y*. *pestis*. These submandibular LN-specific networks could be viable targets for next generation therapeutics.

## Materials and methods

### Aerosolized *Y*. *pestis* exposure to non-human primate (NHP) population

The experimental protocol is described elsewhere [4]. All animal experiments were approved by the Institutional Animal Care and Use Committee (IACUC) at the Walter Reed Army Institute of Research (WRAIR), Silver Spring, MD, and were performed in a facility accredited by the Association for the Assessment and Accreditation of Laboratory Animal Care International (AAALAC).

The adult male African green monkey (*Chlorocebus aethiops*) model used was identified previously as reliable for the study of the pathogenesis of plague [5]. Briefly, the AGMs obtained from the primate colony of WRAIR were 4.8–7.0 kg body weight and determined to be negative for tuberculosis (TB), simian immunodeficiency virus (SIV) and simian retrovirus (SRV). The typical Animal Biosafety Level 3 (ABSL-3) housing conditions and pathogen exposure protocol were reported earlier [4].

A target dose of 100 ± 50 LD_50_ aerosolized *Y*. *pestis* strain CO92 was given to animals that had fasted for the previous 6 hours and were anesthetized using 4 mg/kg Telazol (Fort Dodge Animal Health, Fort Dodge, IA). Our previous report described the methods involved in bacterial inoculation and aerosol delivery [4]. Individual AGMs received *Y*. *pestis* within the range from 0.33 x 10^6^ to 3.55 x 10^6^ CFU via the aerosol particles with an estimated size (mean mass aerosol diameter or MMAD ± geometric standard deviation) of 1.03 μm ± 1.46.

**Fig 1:**
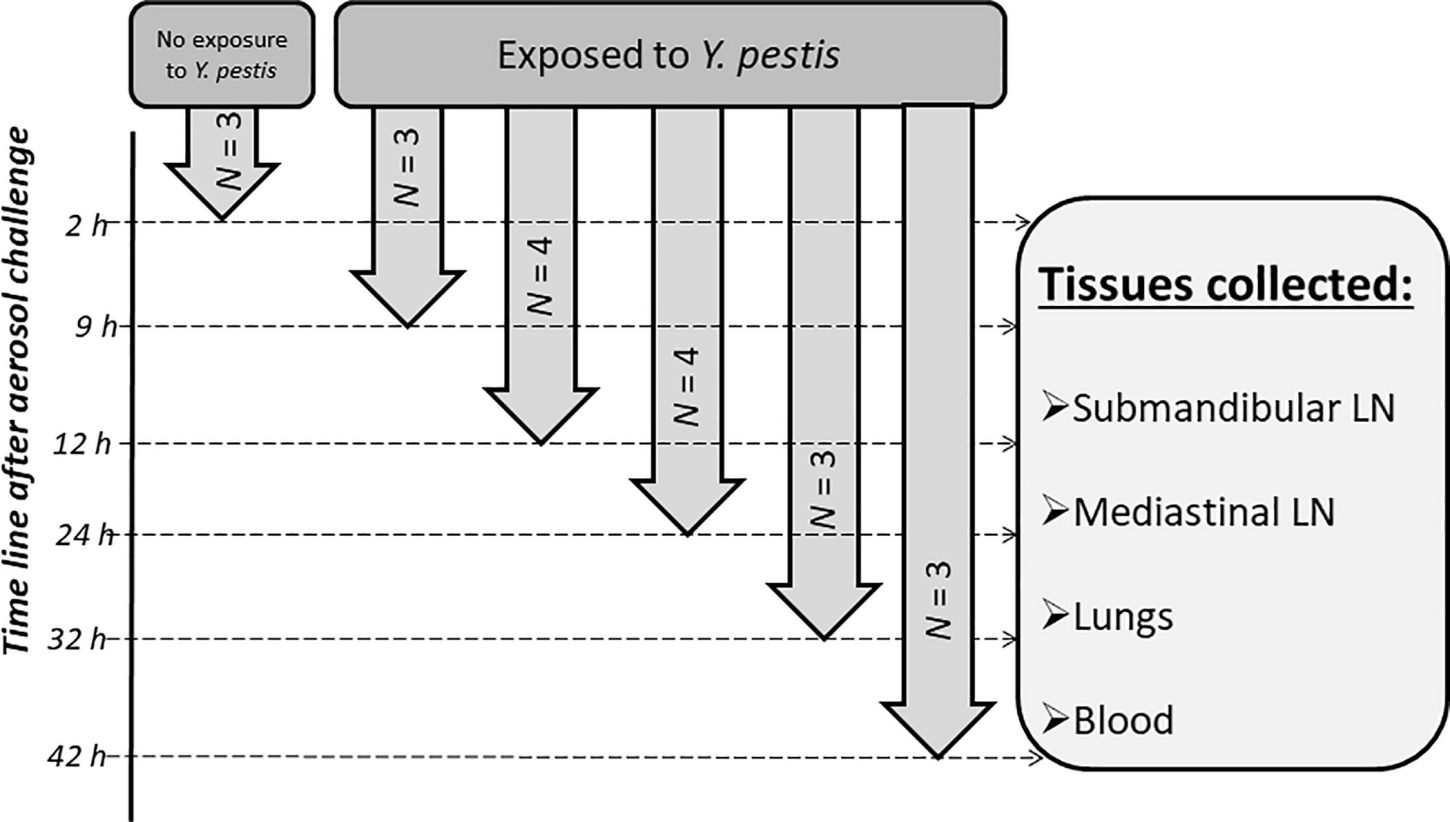
Schematic of experimental design and corresponding organ collection sequence. The timeline is not in scale. *N*: sample size; LN: Lymph node.

A group of three animals was anesthetized but not exposed to *Y*. *pestis*. This control cohort was euthanized 2 h after exposure to sham aerosol. At the 78 h post-exposure time point, we euthanized one *Y*. *pestis*-challenged moribund animal, which was randomly chosen to test the lethality of the given pathogenic load. Between the *Y*. *pestis* exposure and 78 h post-exposure, sub-cohorts (*N* = 3) were sequentially euthanized at 6 h, 9 h, 12 h, 18 h, 24 h, 32 h and 42 h p.i. [4]. The present study is focused on 9 h, 12 h, 24 h, 32 h and 42 h p.i. results. A number of organs, including submandibular LN, mediastinal LN and lungs, were collected from every animal including the controls (Fig 1).

**Fig 2:**
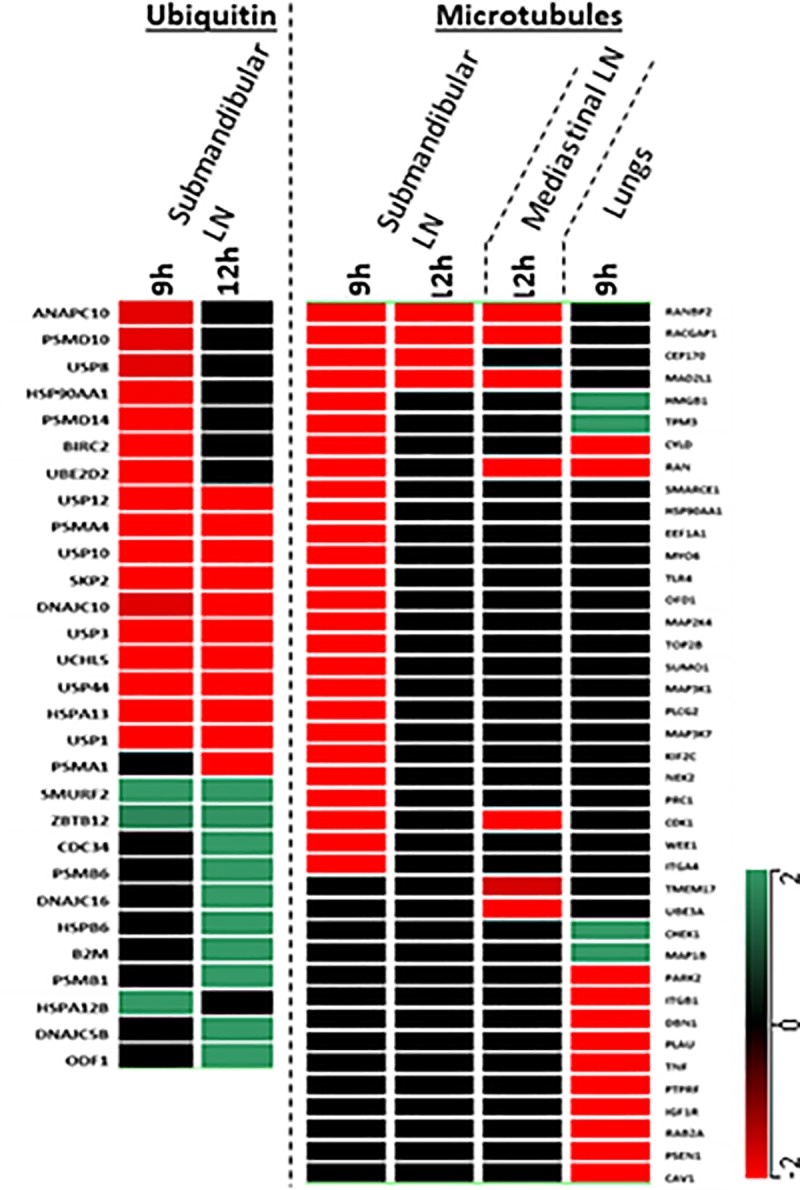
Hierarchical clustering of the genes linked to ubiquitin and microtubules networks. Genes clustered here showed alteration in at least one of the two early time points, 9 h and 12 h p.i. Ubiquitin-associated genes located in submandibular LN are shown in the left hierarchical tree. These genes linked to ubiquitination weren’t expressed in the lungs or mediastinal LN. All of these genes are listed in Table 3, which is in the same order as that in the figure. From the pool of genes linked to microtubule network, only those were mined that ranked in the primary and secondary tiers computed by GIH algorithm. Genes showing early (9 h or 12 h p.i.) alteration in expression are clustered in the right column. All of these genes are listed in Table 4 is in the same order as that in the figure. A scale depicting the range of color corresponding to the gene expression levels is shown at right.

The protocol associated with enumeration of bacterial colonies of blood was discussed earlier [4]. The tissue samples were aseptically collected and portions approximately 1 gm in weight were removed. These portions were placed into properly-labeled, disposable sterile sample bags, heat-sealed, weighed, and placed on ice. These samples were dissociated using a handheld tissue homogenizer (Tissue Tearor, Inc.) for 120 seconds, serially diluted in sterile saline, and cultured for quantitative cultures. Congo red agar plates were incubated at 28°C for 72 h and the CFUs were enumerated based on the geometric mean of CFU/mg of the dilution factor and colony counts (Fig 2, S1 Table).

### Biosample extraction from the organs of interest

Whole organs were submerged in vendor-recommended volumes of TRIzoL Reagent (Life Technologies, Carlsbad, CA). To ensure the TRIzol-emerged samples were non-infectious, these samples were tested for *Y*. *pestis* growth on BHI plates incubated at 28°C for 21 days. Upon observing no growth on the plates, the samples were transferred to BSL2 labs. The organs were homogenized using a handheld sonicator, while keeping the whole system submerged in ice to maintain a low temperature.

RNA was isolated following the protocol outlined for TRIzoL Reagent (Life Technologies, CA). Subsequently, RNA purification was carried out using the Qiagen RNeasy MinElute Cleanup Kit (QIAGEN, Inc., Germantown, MD). RNA quality and quantity were determined using the NanoDrop 2000 spectrometer (Thermo Fisher, Wilmington, DE) and the Agilent 2100 Bioanalyzer (Agilent Technologies, Inc., Santa Clara, CA). Purified RNA was stored at -80°C.

Protein was isolated from the phenol-ethanol supernatant saved during the RNA extraction protocol [23]. Briefly, 100% EtOH was added to precipitate the protein. Protein precipitate was washed with 0.3 M guanidine hydrochloride in 95% EtOH. The protein precipitate was dissolved in 1% SDS, and isolation and purification were completed using the Qiagen protocol.

### cDNA microarray hybridization and post-processing

Agilent Technologies (Agilent Technologies, Inc., CA) was the source of the Rhesus Monkey Oligo Microarray slides containing ~40,000 probes. BioChain’s monkey universal reference RNA (BioChain Institute, Inc., Newark, CA) was labeled with Cy3 dye, and transcribed mRNA (from either control or *Y*. *pestis*-exposed samples) was labeled with Cy5 dye using Agilent’s Quick Amp Labeling Kit (Agilent Technologies, Inc., CA). The cDNA array was co-hybridized using the Cy3- and Cy5-labeled RNAs, the scanned images were visualized and normalized using Feature Extraction (Agilent Technologies, Inc., CA), and the ensuing data were analyzed using GeneSpring software (Agilent Technologies, Inc., CA).

The results are available online (http://www.ncbi.nlm.nih.gov/geo/), GEO ID: GSE101653.

### Protein ELISA

Detection and quantitation of total proteins extracted from all three organs were determined using the Thermo Scientific (Rockford, IL) Pierce BCA Protein Microplate Assay Kit. The microplate reader was set at 562 nm absorbance detection wavelength to determine the quantities of proteins present in the tissue samples.

MyBioSource (San Diego, CA) monkey ubiquitin, and microtubule-associated protein Tau (MAP Tau) and NFκB ELISA kits were used. The vendor-recommended protocol and analytic procedure were used to compare the result with a standard curve to determine the quantitative measure of the expressed proteins.

### Transcriptomic assay validation

The detail of the QuantiGene-Plex protocol was described elsewhere [24]. From Thermo Fisher Scientific (Frederick, MD), we purchased the customized probes of the following NHP genes: ADNP, CYLD and TRIM9 in lungs, and USP12, USP10, SKP2 and PSMA4 for submandibular LN. Beads linked to the probes were hybridized with total RNA selected for individual time points of corresponding tissue types. The hybridization plate was incubated for 22 hours at 54°C, while shaking at 600 RPM in a VorTemp 56 shaking incubator. Post-hybridization, the samples were captured in magnetic separation plates, washed, signal-amplified and scanned using the BioRad BioPlex 100 instrument. The BioPlex instrument settings were sample size 100 ul, timeout 60 seconds, and Bead Events/Bead region 100.

Fluorescent readings from blank wells were subtracted from fluorescent values for each mRNA of interest. These values were then normalized against the geometric mean expression of two control genes for each sample: GAPDH and β-Actin (ACTB). The array data were compared with QuantiGene Plex data in Table 1.

**Table 1:** The list of genes assayed for validation using QuantiGene Plex platform. The high throughput array data and BioPlex/qPCR data are presented in a log_2_ (fold change).Those instances, where the regulations measured by array and qPCR results are in opposite directions were in italics. S6 Fig depicts the bar plots of qPCR results.

Time Course →		9h		12h		24h		32h		42h	
	Gene Name	Array	qPCR	Array	qPCR	Array	qPCR	Array	qPCR	Array	qPCR	Gene Functions
**Lungs**	**ADNP**	-2.50	-6.20	—	—	-2.30	-6.30	-3.00	-0.95	—	—	Provides neuroprotection
	**CYLD**	-1.61	-0.96	—	—	-1.61	-1.80	-2.01	-1.06	—	—	Checkpoint of necrosis [45]
	**TRIM9**	1.80	1.30	-1.91	-1.20	1.61	0.83	—	—	—	—	Linked to proteasomal degradation
**Sub-mandibular LN**	**USP12**	-2.02	-0.27	*-2*.*6*	*0*.*02*	—	—	-2.97	-0.73	-1.90	-1.31	Regulator of T cell homeostasis and cell cycle progression [46, 47]
	**USP10**	-2.26	-0.25	-2.6	-0.60	-2.11	0.82	-2.43	-0.93	-2.04	-2.47	Regulator of DNA damage [48]
	**SKP2**	-2.28	-0.55	-2.42	-0.11	-2.13	1.01	-2.87	-1.17	-1.91	-2.95	Provides a scaffold named SCF for ubiquitin-proteasome activity, immune response, apoptosis and cell signaling [49, 50]
	**PSMA4**	-2.13	-0.90	-2.2	-0.40	-2.47	-0.93	-2.81	-0.44	-2.23	-3.30	Involved with proteasome activity

In addition, the longitudinal expression dynamics of a number of genes in blood were validated using the qPCR assay we published earlier [4]. The expression of genes linked to the functions relevant to this manuscript was reported, including INFγ, XIAP, ELP2, UBE2D1, ADAMTS12, NCR1, SOCS1, NEDD4, ALDH1L1 and IL6.

### Statistical and functional analysis

For all transcriptional and protein analyses, the untreated controls were used as the baseline (or threshold). Transcripts altered at individual time points (moderated *t*-test analysis values of *p* < 0.01 and fold change >|2|) were mined from each organ type. Subsequent network analysis using the significantly altered genes was conducted using the Ingenuity Pathways Analysis platform (IPA, QIAGEN, Inc., CA). Networks meeting the hypergeometric threshold (*p* < 0.05) were considered for subsequent analysis. Functional analysis identified those genes that were associated with various networks of interest. The Molecule Activity Predictor (MAP) toolkit of IPA was used to predict the temporal regulation patterns of networks of interest. The MAP algorithm suggests the state of activation or inhibition of the network of interest by considering the observed expression changes of the molecules associated with this network.

Since a large gene set was identified linked to the microtubule network, it became essential to sort the genes based on their involvement in this biological process. As a result, we were able down-select to obtain those genes which were primarily involved with *Y*. *pestis*-mediated manipulation of the microtubule network. We used the Gene Interaction Hierarchy (GIH) algorithm [25] to sort the genes based on their involvement in the process by which *Y*. *pestis* manipulates microtubule networks. The genes were sorted based on the number of interactions they made with their neighbors within the microtubule networks domain. Candidates ranked within the top 90 percentile were placed into the primary tier, and candidates ranked between 75 and 90 percentile were placed into the secondary tier. The remaining genes that made at least one interaction were pooled within the peripheral group. Some genes did not interact with their neighbors, and they were clustered as the orphan group. Hierarchical clustering of each subgroup was carried out using a Euclidian algorithm.

Protein expression was calculated for every time point using the untreated control samples as the baseline. Fold change cutoff was set at ±1.5. GraphPad Prism was used for visualization and calculation of the statistical significance using Welch’s *t*-test (^#^ *p*<0.1; * *p*< 0.05; ** *p*<0.01).

## Results

AGMs were exposed to aerosolized *Y*. *pestis* and sequentially euthanized at 6 h, 9 h, 12 h, 18 h, 24 h, 32 h and 42 h p.i. Three untreated animals were used as the baseline cohort. Two animals marked for lethality testing died at 72 h-78 h p.i. Post euthanasia, we extracted 13 tissues that included blood, lungs, submandibular LN and mediastinal LN from individual NHPs. [4].

### Early *Y*. *pestis* colonization detected in lungs, submandibular LN and mediastinal LN

S1 Table documented the number of *Y*. *pestis* colonization after the pathogenic exposure. Our previous publication presented the enumeration of bacterial colonies of all the tissue types beyond blood, lungs, submandibular LN and mediastinal LN; in addition, we justified the selection of the threshold [log_10_(1.5 CFU/mg)] and reasons for reporting the sub-threshold number [4].

At 9 h p.i., two animals out of three showed bacterial colonization in the submandibular LN with an average load ten times higher than the threshold. Subsequent time points showed some loss of bacterial load in submandibular LN. A very consistent above-threshold bacterial colonization was observed in the lungs. In mediastinal LN, *Y*. *pestis* colonization that met the threshold was detected at relatively late time point (between 12–24 h p.i). The number of AGMs detected with positive infection in mediastinal LN were found to increase at subsequent time points. As reported earlier, blood showed further delayed detection, with *Y*. *pestis* colonization first observed to meet the threshold value at around 32 h p.i.

### Global transcriptional analysis of the organs of interest

Lungs, submandibular LN and mediastinal LN were selected for protein and transcriptomic analyses. Transcriptional profiles of blood samples were presented earlier, and based on the published results, we analyzed two early (9 h and 12 h), one middle (24 h) and two late time points (32 h and 42 h). In the subsequent text, we refer to 9 h and 12 h p.i. time points together as the early phase of infection.

In principal component analyses (PCA), the unexposed animals showed a clear separation from the *Y*. *pestis*-exposed animals in these three different organs (S1 Fig). The bar graphs of the number of differentially expressed gene transcripts at each time point (S2 Fig do not demonstrate a temporal trend, in contrast to the blood transcriptional dynamics [4].

The cDNA microarray analysis of submandibular LN found that the expression of 1233, 1120, 454, 1653 and 1225 genes was statistically significantly altered at 9 h, 12 h, 24 h, 32 h and 42 h post-exposure, respectively (S2A Fig). At the same significance level, the expression of 284, 346, 186, 92, and 379 genes was altered in mediastinal LN at 9 h, 12 h, 24 h, 32 h and 42 h post-exposure, respectively (S2B Fig). At 9 h, 12 h, 24 h, 32 h and 42 h post-exposure, the expression of 1243, 502, 1201, 855, and 349 genes, respectively, was altered in lungs (S2C Fig).

### Inhibition of ubiquitin indicated in submandibular LN

**Fig 3:**
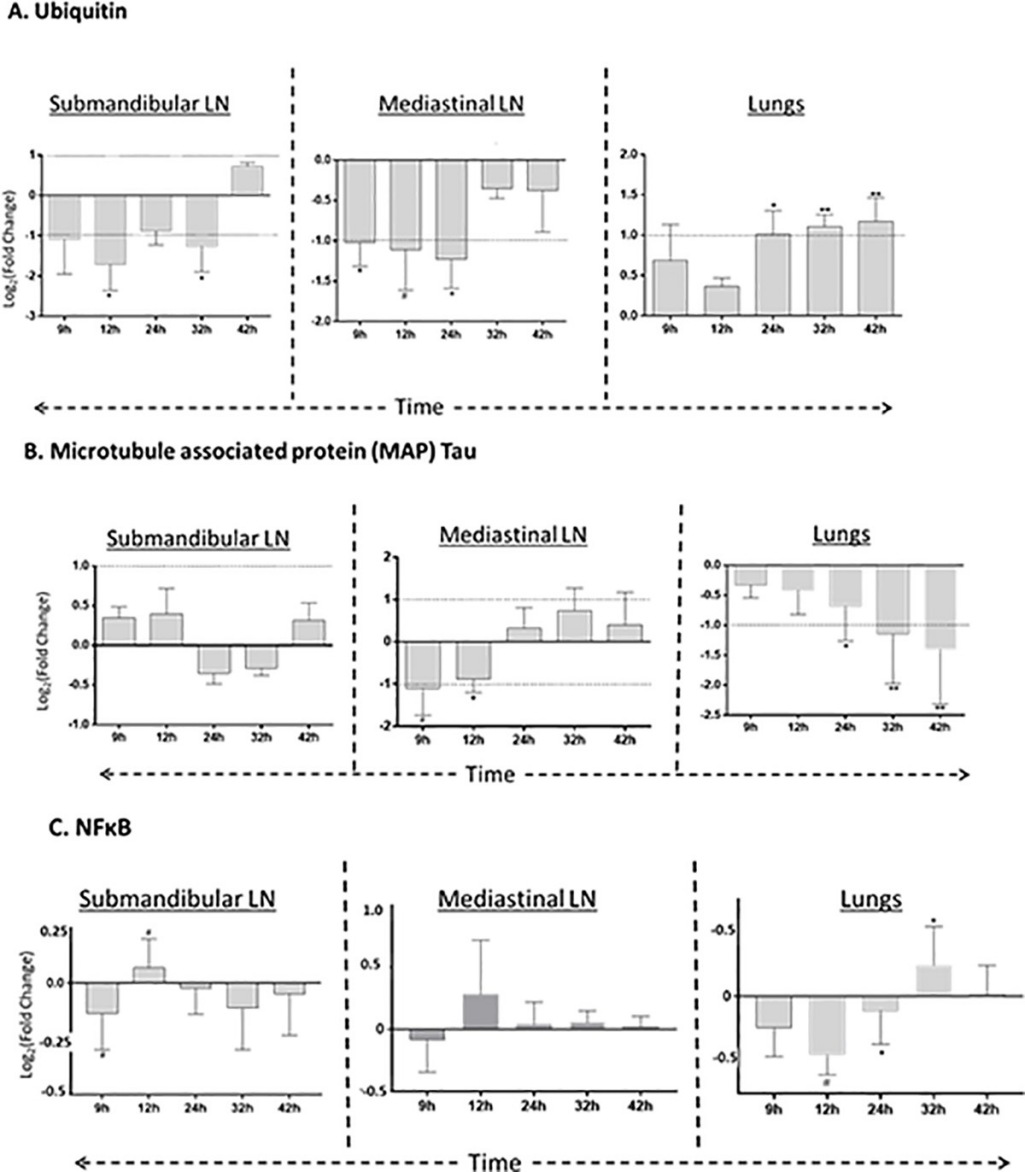
Time and tissue–specific abundance of candidate proteins. (A) Ubiquitin abundance in submandibular LN, Mediastinal LN and Lungs. (B) Microtubule associated protein (MAP) abundance in submandibular LN, Mediastinal LN and Lungs. (C) NFκB abundance in submandibular LN, Mediastinal LN and Lungs. The dotted line parallel to x-axis depicts the cut-off at fold change ±1.5. GraphPad Prism was used for visualization and calculation of the statistical significance using Welch’s *t*-test (^#^ *p*<0.1; * *p*< 0.05; ** *p*<0.01). LN: Lymph node.

Functional analysis of the gene expression data implied that the regulation of the ubiquitin network was significantly altered in submandibular LN (Table 2). S2 Table lists the gene profile enriched for the ubiquitin network. Overall, we found 42 ubiquitination-associated genes with altered expression in the submandibular LN, and the majority (62%) of encoded proteins localized to the cytoplasm. S3 Fig depicts the entire ubiquitin network enriched by the differentially regulated genes; the genes were further segregated based on the locations of the encoded proteins. There were 29 genes linked to the ubiquitin network that showed significant changes in regulation during the early time points (Table 3). Since the objective of the present study is to understand the early pathogenesis, we depicted the hierarchical transcriptomic regulation of two early time points (Fig 3).

**Table 2:** Predicted levels of activation of different biological functions. Upward arrows and downward arrows represent the activation and inhibition, respectively. The ‘x’ represents no change.

**Submandibular LN**					
	**9h**	**12h**	**24h**	**32h**	**42h**
Bacterial infection	↑	X	X	↑	↑
Ubiquitin signal	↓	X	X	↓	↓
Microtubule dynamics	↓	↓	↓	↓	↓
Organization of microtubule	↓	↓	↓	↓	↓
IκB kinase/NFκB cascade	↓	X	↓	↓	↓
TLR signaling	↓	X	X	↓	↓
Apoptosis	↑	↑	X	↑	↑
Inflammatory response	↓	↓	↓	↓	↓
**Lungs**					
	**9h**	**12h**	**24h**	**32h**	**42h**
Bacterial infection	X	X	X	X	X
Ubiquitin signal	X	X	X	X	X
Microtubule dynamics	X	X	X	X	X
Organization of microtubule	X	X	X	X	X
IκB kinase/NFκB cascade	X	X	X	↓	X
TLR signaling	X	X	X	X	X
Apoptosis	↑	↑	↑	X	↑
Inflammatory response	↓	↓	↓	↓	↑
**Mediastinal LN**					
	**9h**	**12h**	**24h**	**32h**	**42h**
Bacterial infection	X	X	X	↑	X
Ubiquitin signal	X	X	X	X	X
Microtubule dynamics	X	↓	↓	↓	↓
Organization of microtubule	X	↓	↓	↓	↓
IκB kinase/NFκB cascade	X	X	X	↓	X
TLR signaling	X	X	X	↓	X
Apoptosis	↑	↑	X	↑	↑
Inflammatory response	↓	↓	↓	↓	↓

**Table 3:** Genes of interest from the ubiquitin network. Genes showing early response for at least two early time points (9 h and 12 h p.i) are listed. Only the significant values (Log_2_ values) are reported.

Gene name	Submandibular LN		Relevant to *Y*. *pestis* pathogenesis
	9 h	12 h	
ANAPC10	-1.8	—	Ubiquitin ligase found susceptible to pathogen [51]
PSMD10	-1.8	—	Involved with proteasome activity
USP8	-1.8	—	Immunomodulator of T cell development [52] and regulator of lysosomal enzymatic activities [53]
HSP90AA1	-1.9	—	Encoded proteins, namely, Hsp70 and Hsp90, are markers of hyperthermia and collectively regulate NFκB-mediated inflammatory responses [54, 55]
PSMD14	-2.0	—	Involved with proteasome activity
BIRC2	-2.5	—	Alters the host response to pathogen [56]
UBE2D2	-2.5	—	An integral part of proteasome system
USP12	-2.0	-2.6	Regulator of T cell homeostasis and cell cycle progression [46, 47]
PSMA4	-2.1	-2.2	Involved with proteasome activity
USP10	-2.3	-2.6	Regulator of DNA damage [48]
SKP2	-2.3	-2.4	Provides a scaffold named SCF for ubiquitin-proteasome activity, immune response, apoptosis and cell signaling [49, 50]
DNAJC10	-2.3	-2.9	Involved in recognizing and degrading misfolded proteins [56]
USP3	-1.8	-2.0	Suppression diminishes the reserve of the hematopoietic stem cell (HSC) and curtails the life span [57]
UCHL5	-2.6	-3.3	Suppression triggers apoptosis and proteotoxicity [58]
USP44	-2.8	-3.3	Regulator of DNA damage [59]
HSPA13	-2.9	-3.8	Involved in removal of denatured or incorrectly folded proteins [60]
USP1	-3.2	-3.7	Suppression causes the arrest of cellular replication and dysregulation of genome stability via controlling cellular senescence [61]
PSMA1	—	-3.1	Involved with proteasome activity
SMURF2	2.1	2.5	Promoter of proteasome-dependent protein degradation [62, 63]
ZBTB12	1.8	2.0	Possibly acts as transcriptional regulation
CDC34	—	2.0	Triggers processive ubiquitination via interlinking with SCF [64]
PSMB6	—	2.9	Early marker of sepsis onset [65]
DNAJC16	—	2.9	Regulator of apoptosis as encoding HSP40 [66]
HSPB6	—	3.1	Regulator of apoptosis and maintains protein homeostasis [67] [68]
B2M	—	3.1	Negative regulator of immune response [69, 70]
PSMB1	—	3.8	Early marker of sepsis onset [65]
HSPA12B	2.3	—	Regulator of apoptosis and neuroinflammation as a distinct family member of HSP40 [71, 72]
DNAJC5B	—	5.6	Regulator of apoptosis as encoding HSP40 [66]
ODF1	—	5.1	A candidate E3 ubiquitin ligase

The MAP algorithm (Table 2) predicted that ubiquitination in submandibular LN was inhibited at 9 h p.i. and remained mostly inhibited across the time points. USP10 and SKP2 remained suppressed across all time points. B2M, ODF1, HSPB6, DNAJC16, DNAJC5B, HSPA12B, SMURF2, PSMB1, PSMB6, CUL1, ZBTB12 and CDC34 showed early up-regulation, whereas HSPA13, HSP90AA1, USP1, USP3, USP8, USP10, USP12, USP44, BIRC2, UCHL5, PSMD10, PSMD14, PSMA4, PSMA1, DNAJC10 and MDM2 showed early down-regulation.

### Cross-organ gene expression profiles predicted early inhibition of microtubule activities

All three major organs at their *Y*. *pestis* ports of entry showed signs of bacterial colonization and concurrent transcriptional changes, suggesting a shift in microtubule activities (Table 2). S3 Table lists the corresponding genomic profiles across these three organs, for which 278 genes were significantly altered and associated with 821 interactions with their neighbors. GIH algorithm [25] -based gene clustering was carried out to down-select those genes which were most critically involved with *Y*. *pestis*-mediated manipulation of microtubule functions. The GIH algorithm found 20 genes ranked in the primary tier that were involved in more than 37% of total interactions. There were 44 genes ranked in a secondary tier. One hundred twenty-nine peripheral genes were involved in at least one interaction with their neighbors. In addition, there were 87 genes with no interactions, defined as orphan genes (S3 Table). S4A and S4B Fig show the distributions of the primary and secondary ranked genes across the three organs, respectively. More than 50% of these genes were located in submandibular LN. MAP analysis showed early inhibition of the microtubule network in submandibular and mediastinal LN (Table 2).

Table 4 lists all the genes related to microtubule functions that ranked in the primary and secondary tiers of GIH and showed early significant alteration in at least one organ of interest. Changes in gene expression of the hierarchical clustered genes listed in Table 4 are shown in Fig 3. Furthermore, we carried out network analysis seeding of only those genes which are documented in Table 4, and the list of networks is shown in S5 Fig.

**Table 4:** Genes of interest from the microtubule network. Listed genes are from the pool ranked in the top tier of the Gene Interaction Hierarchy (GIH). Genes showing early response for at least two early time points (9 h and 12 h p.i.) are listed. Only the significant values (Log_2_ values) are reported.

Gene name	Submandibular LN		Mediastinal LN	Lungs	Relevance to present objective
	9 h	12 h	12 h	9 h	
RANBP2	-2.7	-3.3	-0.5	—	Linked to phosphorylation of histone, an epigenetic marker of cell death [73]
RACGAP1	-3.8	-3.4	-0.5	—	Associated with microtubule binding
CEP170	-3.2	-3.4	—	—	Showed susceptibility to infection facilitating microtubule rearrangements [74]
MAD2L1	-5.0	-4.5	-0.8	—	Critically associated with mitosis
HMGB1	-2.1	—	—	2.1	Modulates phagocytosis, inflammation and cell migration [75]
TPM3	-2.6	—	—	2.1	Linked to apoptosis [76]
CYLD	-2.4	—	—	-1.6	Checkpoint of necrosis [45]
RAN	-2.8	—	-0.6	-2.2	Protein and RNA translocator
SMARCE1	-1.9	—	—	—	Associated with proteostasis and ubiquitin-proteasome [77]
HSP90AA1	-1.9	—	—	—	Promotes maturation, structural alteration and regulation of target proteins [78]
EEF1A1	-1.9	—	—	—	Potentially inhibits viral growth and associated apoptosis [79]
MYO6	-2.0	—	—	—	Contributes to phagocytosis along with microtubules [80]
TLR4	-2.0	—	—	—	Controls energy homeostasis during stress [81]
OFD1	-2.1	—	—	—	Controls centriole, a principal microtubule organizing centers [82]
MAP2K4	-2.1	—	—	—	Directly associated with toll like receptor-mediated pattern recognition [83]
TOP2B	-2.3	—	—	—	Linked to DNA repair [84]
SUMO1	-2.3	—	—	—	Undertakes many ubiquitin-linked functions including proteolysis [85]
MAP3K1	-2.5	—	—	—	Selective activator of JNK-network-mediated apoptosis [86]
PLCG2	-2.5	—	—	—	Linked to immunodeficiency [87]
MAP3K7	-2.6	—	—	—	Recruits JNK, P38, AND NFκB under stress [86]
KIF2C	-2.7	—	—	—	Linked to microtubule assembly [88]
NEK2	-2.8	—	—	—	Controls cell cycle propagation, cell survival and apoptosis [89]
PRC1	-2.9	—	—	—	Controls microtubule architecture [90]
CDK1	-4.6	—	-0.8	—	Controls microtubule dynamics via regulating cell cycle transition and phosphorylation [91]
WEE1	-3.3	—	—	—	Controls immunosurveillance [92]
ITGA4	-4.2	—	—	—	Integral part of T-cell and B-cell based immune mechanism [93]
TMEM17	—	—	-0.4	—	Regulates functions at ciliary transition zone [94]
UBE3A	—	—	-0.5	—	Associated with ubiquitin-proteasome pathway [95]
CHEK1	—	—	—	2.0	Activates DNA repair [96]
MAP1B	—	—	—	1.7	Involved in microtubule assembly and dynamics [97]
PARK2	—	—	—	-1.5	Presents structural fidelity to microtubules [98]
ITGB1	—	—	—	-1.7	Supports microtubule stability [99]
DBN1	—	—	—	-1.7	Crosslink actin and microtubule stability [100]
PLAU	—	—	—	-1.8	Maintain immune homeostasis [101]
TNF	—	—	—	-1.9	Encodes proinflammatory cytokine, which is critically linked to NFκB network
PTPRF	—	—	—	-1.9	Associated with cell adhesion [102]
IGF1R	—	—	—	-1.9	Critically linked to apoptosis [103]
RAB2A	—	—	—	-2.1	Linked to phagocytosis and apoptosis [104]
PSEN1	—	—	—	-2.2	Integral part for proper protein degradation through the autophagosome-lysosome system [105]
CAV1	—	—	—	-3.8	Inhibits the depolymerization of microtubule [106]

### Apoptosis onset and suppression of inflammatory response observed across the organs

S4 Table lists organ-specific genes associated with apoptosis and inflammatory response. The MAP algorithm was used with the IPA platform to compute the regulation of these networks across time points in the three different organs. The trends are shown in Table 2. Interestingly, an early onset of apoptosis and early suppression of inflammatory response were predicted in all three organs. There were 473 apoptotic genes altered in the submandibular LN across all the time points. Among these genes, 204 (42 up- and 162 down-regulated) and 184 (108 up- and 76 down-regulated) were regulated at 9 h and 12 h p.i., respectively. Similarly, 160 inflammatory responding genes were altered in submandibular LN across all of the time points. Among them, 81 (12 up- and 69 down-regulated) and 69 (19 up- and 50 down-regulated) genes were regulated at 9 h and 12 h p.i., respectively.

We also investigated the trend of the toll-like receptor (TLR) signaling networks across the time course (S4 Table). The most prominent responses were in the submandibular LN that included 14 genes linked to the TLR signaling networks. Among them, 9 (4 up- and 5 down-regulated) genes were regulated at 9 h p.i.

### Ubiquitin, MAP Tau and NFκB protein loads were reduced during the early phase of infection

**Fig 4:**
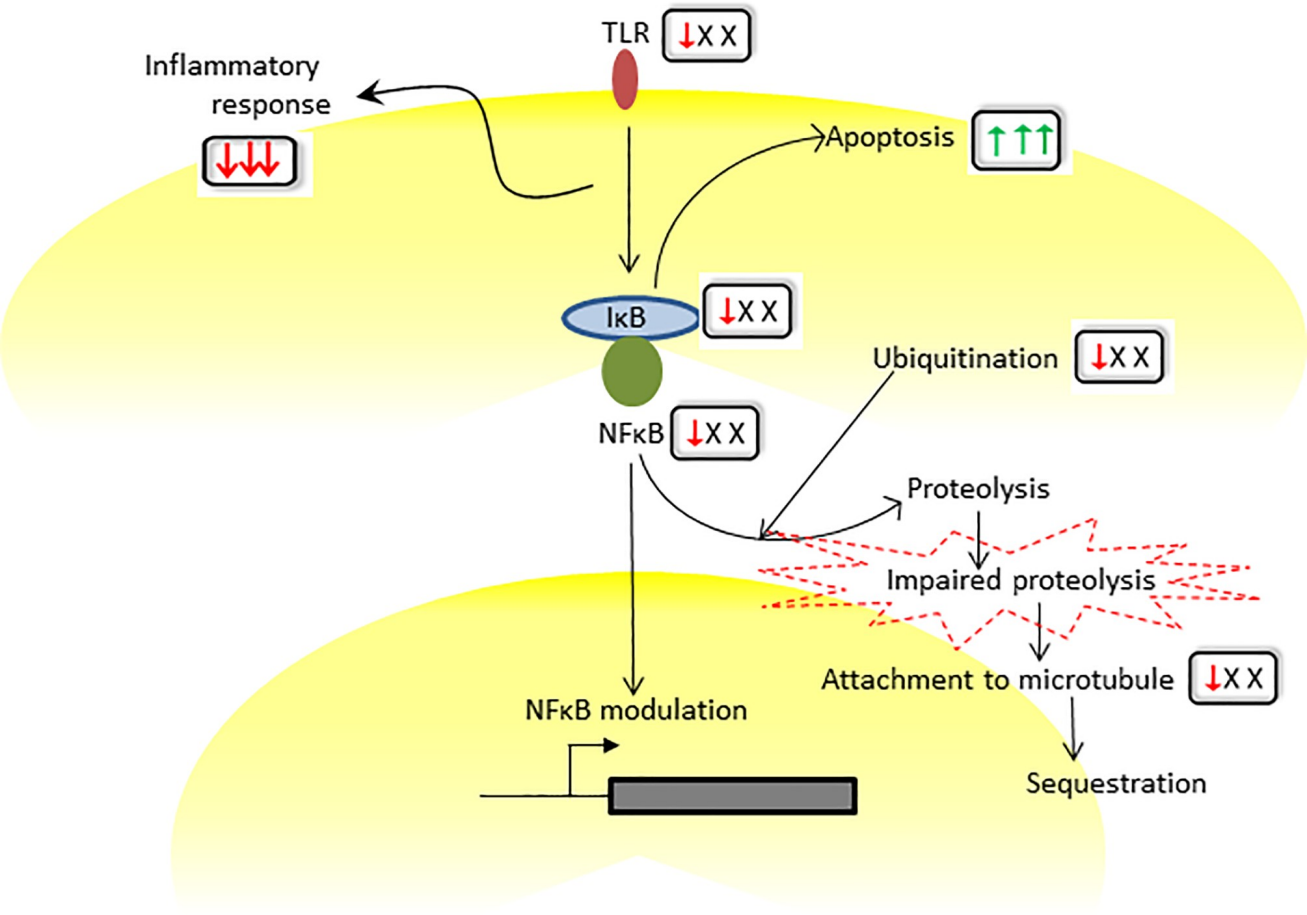
A pictorial representation of the cluster of networks that became susceptible to *Y*. *pestis* at 9 h p.i. The box adjacent to the gene/function represents the corresponding predicted level of regulations in submandibular LN, lungs and mediastinal LN, respectively at 9 h p.i. The predicted regulations of ubiquitin, microtubules and NFκB were confirmed by investigating the time and tissue specific abundance of candidate proteins. The green upward arrow and red downward arrow represent the activation and inhibition, respectively. The ‘x’ stands for no change.

Fig 4 depicts the abundance of the (Fig 4A) ubiquitin, (Fig 4B) MAP Tau and (Fig 4C) NFκB proteins in the three organs. Ubiquitin abundance was mostly suppressed in the submandibular and mediastinal LNs; the suppressed loads of ubiquitin met the threshold of detection at the early phase of infection. In contrast, the abundance of ubiquitin consistently increased in the lungs across the time range.

MAP Tau protein abundance in the submandibular LN never met the threshold level. Decreased MAP Tau expression in the mediastinal LN crossed the threshold of detection only at the early time points. In the lungs, MAP Tau protein abundance became increasingly suppressed with time, crossing the detection threshold at 32 h p.i.

The submandibular LN had a low abundance of NFκB protein. The level of reduction at 9 h p.i. was nearly significant. Likewise, the load of NFκB protein remained at the sub-threshold level in mediastinal LN. In the lungs, the load of NFκB protein remained less than baseline level during the early phase of infection (from 9 h to 24 h p.i).

## Discussion

A recent assessment of the challenges faced by the plague vaccine development program pointed out the scarcity of NHP data in the field of understanding plague pathogenesis [26]. Mizel et al., explored a multi-organ study using a NHP model where the assays were limited to an antigen-specific humoral immune response [27]. To our knowledge, our previous study [4] was the first to interrogate the global transcriptomic profile of NHP blood to understand the early pathogenesis of pneumonic plague. While the majority of the published literature used rodents to understand the temporal progression of *Y*. *pestis* [28–30], we deemed it a logical step to investigate NHPs since they are a species that is phylogenetically and physiologically near to humans. However, it is important to note that the study of *Yersinia* pathogenesis using rodent models has particular value, since these species are the natural hosts of this pathogen. Furthermore, the present study derived incentive for its focus from many rodent studies that suggested the critical roles of lungs and adjacent lymph nodes in the host response to *Yersinia* [31, 32], including a recent study that reported the particular affinity of *Y*. *pestis* in colonizing lymph systems post-subcutaneous infection, as detected using a whole body bioluminescent tracing protocol [33].

Like humans, AGMs are good at hiding their physical discomforts until very late [4, 5]. No striking clinical symptoms were noticed until one day prior to their deaths. The cornerstone of our findings was that many immunological and host-response networks were perturbed in tissues much earlier than the bacterial colonization was observed in those tissues. Significant colonization of *Y*. *pestis* in blood was noted at 32 h p.i. although sub-threshold bacterial colonization were observed starting from 12 h p.i. (S1 Table). In contrast, lungs (Fig 2A, S1 Table) and submandibular LN (Fig 2B, S1 Table) manifested significant colonization of *Y*. *pestis* by 9 h p.i. These organs are located near the respiratory tract. Hence, early colonization of *Y*. *pestis* in these organs is rather expected since the NHPs were intranasally challenged with *Y*. *pestis*. Comprehension of those molecular events that take place in these organs is likely to elucidate the early host-pathogen interactions that ultimately failed to contain *Y*. *pestis*. Notably, mediastinal LN are in close proximity of the lungs and showed colonization between 12–24 h p.i. (Fig 2C, S1 Table), whereas the transcriptomic changes in mediastinal LN were noted starting at 9 h p.i. Hence, both blood and mediastinal LN underwent molecular perturbations before bacterial colonization was observed, which showcases the advantage of determining molecular markers as the early sign of infection.

Despite the anatomic differences between the lymph nodes and lungs, the trends of their transcriptomic responses to *Y*. *pestis* infection were very similar as suggested by their PCA plots (S1 Fig). Among all of these cases, the largest transcriptomic shift from the pre-infection state happened at the earliest time point of 9 h p.i. In comparison, the molecular shifts in the subsequent time points appeared negligible. PCA plots displayed a large displacement and essentially conglomeration of the post-infection time points away from the pre-infection time point for transcriptomic regulation. Therefore, one can posit that the impact of *Y*. *pestis* assault was immediate, and this early impact potentially caused the maximum damage in these organs.

Limitations of this study included using the Rhesus Monkey Oligo array to probe the AGM transcriptomic profile. The small sample size per time point also compromised the statistical power of the study. To mitigate this risk, a longitudinal study was designed which was held together by transcriptomics-proteomics analyses. The untargeted transcriptomic investigation was streamlined to focus on the networks of interest, and stringent statistical thresholds were in place to deliver a robust molecular interpretation. Time-specific NHP controls at every time point were replaced with a more cost-efficient solution: a baseline control was euthanized at the beginning of the study. The high throughput transcriptomic results were validated by targeted proteomics and transcriptomic data. The strict regulations imposed on BSL3 labs prohibited us from isolating cells critical for adaptive and innate immunity. Hence, the present study lacks cell-specific data and histopathology data.

### Early highjack of the ubiquitination process in the submandibular LN potentially compromises proteolytic activity

Ubiquitin plays a versatile role in cell biology, and facilitating the host cellular defense is one of its key roles [8, 9]. Our previous study showed early activation of the ubiquitin network in the blood transcriptomic profile [4]. The ubiquitin network became suppressed at the transcriptional level in the submandibular LN by 9 h p.i. In concert, a low abundance of ubiquitin was observed in submandibular LN. The consequences could be far reaching since the efficacy of the host immune mechanism essentially depends on the proteasome-mediated disposal of ubiquitinated proteins [34]. Ubiquitins play critical roles during infection by supporting the antigen-presenting cells involved in host immunity and in activation of the NFκB family of transcription factors. Ubiquitins also participate in the degradation of whole organelles and large protein aggregates via autophagy [34], which is discussed in a later section in the context of microtubule function.

Existing literature has identified YopJ, a virulence factor of Yersinia, as an efficient deubiquitinating agent [35] enabling deactivation of NFκB-mediated host defense mechanisms. Our study found a set of proteasome-encoding genes that included PSMA1, PSMD10, PSMD14 and PSMA4, that were all down-regulated during the early phase of infection. Our functional analyses predicted that the early suppression of the toll-like receptor network and the IκB kinase/NFκB cascade network (Fig 4) potentially triggered an early deactivation of the NFκB signal. In support of this hypothesis, we found a nearly significant reduced load of NFκB protein in submandibular LN at 9 h p.i. Impaired proteocatalytic activities were further showcased by a group of down-regulated ubiquitin-linked genes, USP1, USP3, USP8, USP10, USP10, USP12 and USP44, that encode proteins associated with peptidase. Hence, there is an early indication of dysregulated ubiquitin-proteasome activity caused by *Y*. *pestis* infection.

Notably, the ubiquitin network was not significantly perturbed in mediastinal LN and lungs, and this essentially highlights the complex inter-organismal relationship [36]. Nevertheless, the trend was very clear. In submandibular LN, the ubiquitin networks remained inhibited during the early and late phases of infection (Table 2). By integrating this information with other networks closely connected to ubiquitin, such as microtubule activities, inflammation, and apoptosis, we found a more compelling picture of the pathogenesis of plague.

### Early dysregulation of the microtubule network potentially limited the protein sequestration process

Typically, pathogens trigger the microtubular activities in order to enhance the local spread of infections [16]. Alternatively, destabilization of host microtubules results in impairment of the autophagosome, permitting an easy access to the cytoplasm, and ultimately, evasion of the host defense. Our study showed early down-regulation of genes linked to microtubule networks in submandibular LN, mediastinal LN and lungs. The HSP90AA1 gene encoding the chaperone protein for MAP Tau was down-regulated at 9 h p.i. In concert, the load of MAP Tau protein, a critical constituent of stable microtubules [37], was reduced during the early phase of infection in all three organs.

In the event of proteosomal impairment, host cells are essentially compelled to use alternative routes to dispose of pathogens. Hence, the pathogen or its derivative is trafficked to aggresomes via retrograde transportation undertaken by microtubules. Aggresomes are controlled by GTPase-mediated vesicular trafficking and are eventually sequestered by autophagy [38]. Thus in the event of compromised microtubular activities, autophagy becomes dysfunctional and essentially facilitates the proliferation of pathogens.

The scientific literature suggested a somewhat contrasting picture. *Y*. *pestis* was found to block autophagosomes *in vitro* by altering the functions of AKT, AMPK and p53 signaling [39]. An *in vivo* study observed a slightly different mechanism, where *Y*. *pestis* evaded the host defense by entering mouse macrophages and continuing to proliferate within the autophagosomes [40]. The NHP model revealed a number of genes associated with autophagy, such as HSP90AA1, HSPA13, MYO6 and HMGB1 that were down-regulated during the 9 h-12 h p.i. period. In contrast, LMTOR3, the gene encoding a protein responsible for mTOR signal activation, was down-regulated during the early phase of infection, indicating positive regulatory effects of autophagy. As time went by, an increasing number of genes that potentially regulate autophagy were found down-regulated. In particular, a large set of genes associated with AMPK-mediated autophagy were down-regulated, including PK1A, PRKAA1, PRKACB, CREM (all at 24 h p.i); CREB3L3, PK1A, PRKAA1 (all at 32 h p.i.); and CREB3L3, CREB, PK1A, PRKAA1, PRKACB (all at 42 h p.i.). In addition, a number of genes associated with GTPase activities, which included GIMAP2, RACGAP1, AGBL5 and RAP1GDS1, were down-regulated during the early phase of infection. Overall, a temporal trend was established, highlighting a possible mechanism adopted by *Y*. *pestis* to evade autophagy.

The functional analysis of the genes critically associated with microtubule activities (i.e., the genes enriching the two top tiers classified by the GIH algorithm) found that these genes co-enriched the ubiquitin network, TLR signal, and apoptosis (S5 Fig). This result is supported by the emerging evidence that interlinks microtubule-protein networks and cell death [41]. Our results possibly indicated the existence of a parallel apoptotic mechanism induced by *Y*. *pestis* that further weakened the host defense.

### Immunosuppression synchronized with the early onset of apoptosis results in the comprehensive dysregulation of the host defense

Our previous study indicated that *Y*. *pestis* triggered the onset of apoptosis in blood [4]. S5 Fig highlights the possible cross-talk between apoptosis with the ubiquitin-microtubule network. Interrogation of the three organs also revealed that apoptosis may have been triggered during the early phase of infection (Fig 4). The early trend of apoptotic risk was manifested during 9 h-12 h p.i. as nearly 60% of all of the apoptotic genes were altered during this early phase of *Y*. *pestis* infection. The early signal of apoptosis in submandibular LN was synchronized with putative inhibition of TLR signal (Fig 4). This observation is particularly interesting in light of a recent study that reported *Yersinia* species showed more virulent pathogenesis in TLR-deficient mice [42].

We found that suppressed TLR signaling and the early highjack of ubiquitin-microtubule networks were potentially synchronized with the suppression of the host inflammatory response. Although the genes encoding the pro-inflammatory cytokines such as IL-18 were up-regulated in submandibular LN, the overall inflammatory response was putatively suppressed during the early phase of infection. As the time went by, an increasing number genes encoding pro-inflammatory cytokines such as TGFA and IL-36 and anti-inflammatory cytokines such as IL-17 became down-regulated at the transcriptomic level.

### Conclusion

Over time, the pathogenesis of *Y*. *pestis* involves multiple organs and affects many biological functions. To understand this process, we explored the conceivable relationships among the seemingly distinct networks. Aligned to the emerging hypothesis that complex disease biology is essentially driven by clusters of networks [18], we hereby showed the interconnectivity among a set of networks which was perturbed in a synchronized fashion across multiple organs during a pre-symptomatic time period. The present study added a time-resolved perspective to many of those molecular events, such as ubiquitin and microtubule functions that were reported previously in the context of *Y*. *pestis* pathogenesis [13, 14, 35, 39, 40]. In fact, our previous publication [4] upheld ubiquitin and microtubule functions as two major networks potentially manipulated by *Y*. *pestis*.

Submandibular LN emerged as a potential target organ for early intervention. Past records have shown high activity in submandibular LN in the event of aerosol challenge [43, 44]. A particular time lag between submandibular LN and blood molecular profiles was found. For instance, TLR signal was activated in blood during the early phase of infection, and *Y*. *pestis* was able to gradually dysregulate this network. The ubiquitin network remained active across the time course in blood. However, both of these networks were dysregulated in the early phase of *Y*. *pestis* infection in submandibular LN. Thereby our study revealed how some of the key organs potentially cross-talk during the time course of pathogenesis. For an opportunistic pathogen like *Y*. *pestis*, which proliferates rapidly and is capable of turning the key host defense mechanisms to its favor, it is essential to target the appropriate organs and molecular networks to effectively confront and counter the pathogen. Our study identified submandibular LN as an early site for the host immune system to encounter pathogens, and we mined some potential pathways that were impaired by *Y*. *pestis* to create its permissive niche. A carefully designed *in vitro* study that is preferably based on synthetic organ clusters could be used to validate the present multi-organ based analysis. A bio bank obtained from well-regulated clinical efforts would be another valuable source for validating the results. This knowledge can potentially drive the strategies for next generation therapeutics.

## Supporting information

S1 Fig Principal component analysis (PCA) of the global transcriptomic expression of three organs of interest. Each circle is labeled by the corresponding time points. (A) Submandibular LN. (B) Lungs. (C) Mediastinal LN.
(TIF)

S2 Fig Bar plot showing the number of gene transcripts significantly altered in tissues of interest. (A) submandibular lymph node, (B) mediastinal lymph node and (C) lungs. The shaded bar represents up regulated and clear bar represents down regulated probes.
(TIF)

S3 Fig Gene network associated with ubiquitination in submandibular LN. The diagram depicts the cellular locations of the proteins encoded by the genes enriching the ubiquitin network. The temporal expression of individual genes are shown under each gene symbol. To note, there was no ubiquitin-associated gene significantly altered at 24 h p.i.; hence gene expression at 9 h, 12 h, 32 h and 42 h are shown.
(TIF)

S4 Fig Cross-organ distribution genes related to microtubule network; the genes ranked by Gene Interaction Hierarchy (GIH). (A) Primary tier. (B) Secondary tier. The pie chart shows the distribution of genes across three organs, and the corresponding hierarchical matrix shows the temporal pattern of transcriptional expression of these genes in three organs. A scale depicting the range of color corresponding to the gene expression levels is shown at right. Each row corresponds to one particular gene. LN: Lymph node.
(TIF)

S5 Fig Canonical networks significantly enriched with the genes listed in Table 4. The top axis represents the ratio of the genes altered by Y. pestis infection and the entire gene list of this canonical network. The bottom axis represents–log(p value), where the p value represents the enrichment factor calculated by the hypergeometric test.
(TIF)

S6 Fig Bar plots of qPCR results. For each gene, 5 time points were represented with error bars. The assay results showing zero fold changes were marked as *.
(TIF)

S1 Table The longitudinal profile of bacterial loads in tissues of interests: Lungs, Submandibular LN, Mediastinal LN and Blood. (XLSX)

S2 Table Gene list enriching ubiquitin network in submandibular LN. (XLSX)

S3 Table Gene list enriching microtubule network mined from three organs of interest. (XLSX)

S4 Table Gene list linked to other relevant networks mined from three organs of interest. (XLSX)

## References

[1] Tourdjman M, Ibraheem M, Brett M, Debess E, Progulske B, Ettestad P, et al Misidentification of Yersinia pestis by automated systems, resulting in delayed diagnoses of human plague infections—Oregon and New Mexico, 2010–2011. Clinical infectious diseases: an official publication of the Infectious Diseases Society of America. 2012;55(7):e58–60. 10.1093/cid/cis578 . 22715170

[2] Pechous RD, Sivaraman V, Stasulli NM, Goldman WE. Pneumonic Plague: The Darker Side of Yersinia pestis. Trends in microbiology. 2016;24(3):190–7. 10.1016/j.tim.2015.11.008 . 26698952

[3] Perry RD, Fetherston JD. Yersinia pestis—etiologic agent of plague. Clinical microbiology reviews. 1997;10(1):35–66. 8993858 10.1128/cmr.10.1.35 PMC172914

[4] Hammamieh R, Muhie S, Borschel R, Gautam A, Miller SA, Chakraborty N, et al Temporal Progression of Pneumonic Plague in Blood of Nonhuman Primate: A Transcriptomic Analysis. PloS one. 2016;11(3):e0151788 10.1371/journal.pone.0151788 27003632 PMC4803270

[5] Layton RC, Brasel T, Gigliotti A, Barr E, Storch S, Myers L, et al Primary pneumonic plague in the African Green monkey as a model for treatment efficacy evaluation. Journal of medical primatology. 2011;40(1):6–17. 10.1111/j.1600-0684.2010.00443.x 20722770 PMC2991483

[6] Ashida H, Kim M, Sasakawa C. Exploitation of the host ubiquitin system by human bacterial pathogens. Nature reviews Microbiology. 2014;12(6):399–413. 10.1038/nrmicro3259 . 24801936

[7] Asrat S, Davis KM, Isberg RR. Modulation of the host innate immune and inflammatory response by translocated bacterial proteins. Cellular microbiology. 2015;17(6):785–95. 10.1111/cmi.12445 25850689 PMC4632489

[8] Popovic D, Vucic D, Dikic I. Ubiquitination in disease pathogenesis and treatment. Nature medicine. 2014;20(11):1242–53. 10.1038/nm.3739 . 25375928

[9] Collins CA, Brown EJ. Cytosol as battleground: ubiquitin as a weapon for both host and pathogen. Trends in cell biology. 2010;20(4):205–13. 10.1016/j.tcb.2010.01.002 . 20129784

[10] LaRock CN, Cookson BT. The Yersinia virulence effector YopM binds caspase-1 to arrest inflammasome assembly and processing. Cell host & microbe. 2012;12(6):799–805. 10.1016/j.chom.2012.10.020 23245324 PMC3703949

[11] Chung LK, Philip NH, Schmidt VA, Koller A, Strowig T, Flavell RA, et al IQGAP1 is important for activation of caspase-1 in macrophages and is targeted by Yersinia pestis type III effector YopM. mBio. 2014;5(4):e01402–14. 10.1128/mBio.01402-14 24987096 PMC4161239

[12] Mukherjee S, Keitany G, Li Y, Wang Y, Ball HL, Goldsmith EJ, et al Yersinia YopJ acetylates and inhibits kinase activation by blocking phosphorylation. Science. 2006;312(5777):1211–4. 10.1126/science.1126867 . 16728640

[13] Cowan C, Jones HA, Kaya YH, Perry RD, Straley SC. Invasion of epithelial cells by Yersinia pestis: evidence for a Y. pestis-specific invasin. Infection and immunity. 2000;68(8):4523–30. 10899851 10.1128/iai.68.8.4523-4530.2000 PMC98364

[14] Aepfelbacher M, Zumbihl R, Heesemann J. Modulation of Rho GTPases and the actin cytoskeleton by YopT of Yersinia. Current topics in microbiology and immunology. 2005;291:167–75. . 15981463 10.1007/3-540-27511-8_9

[15] Olzmann JA, Li L, Chudaev MV, Chen J, Perez FA, Palmiter RD, et al Parkin-mediated K63-linked polyubiquitination targets misfolded DJ-1 to aggresomes via binding to HDAC6. The Journal of cell biology. 2007;178(6):1025–38. 10.1083/jcb.200611128 17846173 PMC2064625

[16] Radhakrishnan GK, Splitter GA. Modulation of host microtubule dynamics by pathogenic bacteria. Biomolecular concepts. 2012;3(6):571–80. 10.1515/bmc-2012-0030 23585820 PMC3625037

[17] Consortium GT. Erratum: Genetic effects on gene expression across human tissues. Nature. 2017 10.1038/nature25160 . 29022597 PMC5776756

[18] Barabasi AL, Gulbahce N, Loscalzo J. Network medicine: a network-based approach to human disease. Nature reviews Genetics. 2011;12(1):56–68. 10.1038/nrg2918 21164525 PMC3140052

[19] Nakamura S, Hayashidani H, Iwata T, Namai S, Une Y. Pathological changes in captive monkeys with spontaneous yersiniosis due to infection by Yersinia enterocolitica serovar O8. Journal of comparative pathology. 2010;143(2–3):150–6. 10.1016/j.jcpa.2010.01.017 20207365

[20] Zdolec N, Dobranić V, Filipović I. Prevalence of Salmonella spp. and Yersinia enterocolitica in/on tonsils and mandibular lymph nodes of slaughtered pigs. Folia microbiologica. 2015;60(2):131–5. 10.1007/s12223-014-0356-9 25293839

[21] John ALS, Ang WG, Huang M-N, Kunder CA, Chan EW, Gunn MD, et al S1P-Dependent trafficking of intracellular yersinia pestis through lymph nodes establishes Buboes and systemic infection. Immunity. 2014;41(3):440–50. 10.1016/j.immuni.2014.07.013 25238098 PMC4440548

[22] Chung LK, Bliska JB. Yersinia versus host immunity: how a pathogen evades or triggers a protective response. Current opinion in microbiology. 2016;29:56–62. 10.1016/j.mib.2015.11.001 26638030 PMC4755919

[23] Likhite N, Warawdekar UM. A unique method for isolation and solubilization of proteins after extraction of RNA from tumor tissue using trizol. Journal of biomolecular techniques: JBT. 2011;22(1):37–44. 21455480 PMC3059540

[24] Muhie S, Gautam A, Chakraborty N, Hoke A, Meyerhoff J, Hammamieh R, et al Molecular indicators of stress-induced neuroinflammation in a mouse model simulating features of post-traumatic stress disorder. Translational psychiatry. 2017;7(5):e1135 Epub 2017/05/24. 10.1038/tp.2017.91 . 28534873 PMC5534959

[25] White TE, Ford BD. Gene Interaction Hierarchy Analysis Can Be an Effective Tool for Managing Big Data Related to Unilateral Traumatic Brain Injury. In: Kobeissy FH, editor. Brain Neurotrauma: Molecular, Neuropsychological, and Rehabilitation Aspects. Frontiers in Neuroengineering. Boca Raton (FL)2015. 26269909

[26] Verma SK, Tuteja U. Plague Vaccine Development: Current Research and Future Trends. Frontiers in immunology. 2016;7:602 10.3389/fimmu.2016.00602 28018363 PMC5155008

[27] Mizel SB, Graff AH, Sriranganathan N, Ervin S, Lees CJ, Lively MO, et al Flagellin-F1-V fusion protein is an effective plague vaccine in mice and two species of nonhuman primates. Clinical and vaccine immunology: CVI. 2009;16(1):21–8. 10.1128/CVI.00333-08 18987167 PMC2620661

[28] Lathem WW, Crosby SD, Miller VL, Goldman WE. Progression of primary pneumonic plague: a mouse model of infection, pathology, and bacterial transcriptional activity. Proceedings of the National Academy of Sciences of the United States of America. 2005;102(49):17786–91. 10.1073/pnas.0506840102 16306265 PMC1308902

[29] Agar SL, Sha J, Foltz SM, Erova TE, Walberg KG, Parham TE, et al Characterization of a mouse model of plague after aerosolization of Yersinia pestis CO92. Microbiology. 2008;154(Pt 7):1939–48. 10.1099/mic.0.2008/017335-0 . 18599822

[30] Galindo CL, Moen ST, Kozlova EV, Sha J, Garner HR, Agar SL, et al Comparative Analyses of Transcriptional Profiles in Mouse Organs Using a Pneumonic Plague Model after Infection with Wild-Type Yersinia pestis CO92 and Its Braun Lipoprotein Mutant. Comparative and functional genomics. 2009;2009:914762 10.1155/2009/914762 20145715 PMC2817383

[31] Sivaraman V, Pechous RD, Stasulli NM, Eichelberger KR, Miao EA, Goldman WE. Yersinia pestis activates both IL-1β and IL-1 receptor antagonist to modulate lung inflammation during pneumonic plague. PLoS pathogens. 2015;11(3):e1004688 10.1371/journal.ppat.1004688 25781467 PMC4363893

[32] Zimbler DL, Schroeder JA, Eddy JL, Lathem WW. Early emergence of Yersinia pestis as a severe respiratory pathogen. Nature communications. 2015;6:ncomms8487. 10.1038/ncomms8487 PMC4491175 26123398

[33] Zhou Y, Zhou J, Ji Y, Li L, Tan Y, Tian G, et al Bioluminescent tracing of a Yersinia pestis pCD1+-mutant and Yersinia pseudotuberculosis in subcutaneously infected mice. Microbes and infection. 2018;20(3):166–75. 10.1016/j.micinf.2017.11.005 29180033

[34] Kim M, Otsubo R, Morikawa H, Nishide A, Takagi K, Sasakawa C, et al Bacterial effectors and their functions in the ubiquitin-proteasome system: insight from the modes of substrate recognition. Cells. 2014;3(3):848–64. 10.3390/cells3030848 25257025 PMC4197628

[35] Zhou H, Monack DM, Kayagaki N, Wertz I, Yin J, Wolf B, et al Yersinia virulence factor YopJ acts as a deubiquitinase to inhibit NF-kappa B activation. The Journal of experimental medicine. 2005;202(10):1327–32. 10.1084/jem.20051194 16301742 PMC2212976

[36] Consortium GT, Laboratory DA, Coordinating Center -Analysis Working G, Statistical Methods groups-Analysis Working G, Enhancing Gg, Fund NIHC, et al Genetic effects on gene expression across human tissues. Nature. 2017;550(7675):204–13. 10.1038/nature24277 . 29022597 PMC5776756

[37] Dehmelt L, Halpain S. The MAP2/Tau family of microtubule-associated proteins. Genome biology. 2005;6(1):204 10.1186/gb-2004-6-1-204 15642108 PMC549057

[38] Huang J, Brumell JH. Bacteria-autophagy interplay: a battle for survival. Nature reviews Microbiology. 2014;12(2):101–14. 10.1038/nrmicro3160 . 24384599 PMC7097477

[39] Alem F, Yao K, Lane D, Calvert V, Petricoin EF, Kramer L, et al Host response during Yersinia pestis infection of human bronchial epithelial cells involves negative regulation of autophagy and suggests a modulation of survival-related and cellular growth pathways. Frontiers in microbiology. 2015;6:50 10.3389/fmicb.2015.00050 25762983 PMC4327736

[40] Pujol C, Bliska JB. Turning Yersinia pathogenesis outside in: subversion of macrophage function by intracellular yersiniae. Clinical immunology. 2005;114(3):216–26. 10.1016/j.clim.2004.07.013 . 15721832

[41] Parker AL, Kavallaris M, McCarroll JA. Microtubules and their role in cellular stress in cancer. Frontiers in oncology. 2014;4:153 10.3389/fonc.2014.00153 24995158 PMC4061531

[42] Choi JA, Jeong YJ, Kim JE, Kang MJ, Kim JC, Oh SM, et al A role for Toll-like receptor 4 in the host response to the lung infection of Yersinia pseudotuberculosis in mice. Comparative immunology, microbiology and infectious diseases. 2016;44:54–60. 10.1016/j.cimid.2016.01.001 . 26851596

[43] Zaucha GM, Pitt LM, Estep J, Ivins BE, Friedlander AM. The pathology of experimental anthrax in rabbits exposed by inhalation and subcutaneous inoculation. Archives of pathology & laboratory medicine. 1998;122(11):982–92. . 9822127

[44] Beyer W, Turnbull PC. Anthrax in animals. Molecular aspects of medicine. 2009;30(6):481–9. 10.1016/j.mam.2009.08.004 . 19723532

[45] O'Donnell MA, Perez-Jimenez E, Oberst A, Ng A, Massoumi R, Xavier R, et al Caspase 8 inhibits programmed necrosis by processing CYLD. Nature cell biology. 2011;13(12):1437–42. 10.1038/ncb2362 22037414 PMC3229661

[46] Jahan AS, Lestra M, Swee LK, Fan Y, Lamers MM, Tafesse FG, et al Usp12 stabilizes the T-cell receptor complex at the cell surface during signaling. Proceedings of the National Academy of Sciences of the United States of America. 2016;113(6):E705–14. 10.1073/pnas.1521763113 26811477 PMC4760780

[47] Tang LJ, Li Y, Liu YL, Wang JM, Liu DW, Tian QB. USP12 regulates cell cycle progression by involving c-Myc, cyclin D2 and BMI-1. Gene. 2016;578(1):92–9. 10.1016/j.gene.2015.12.006 . 26680102

[48] Zhang M, Hu C, Tong D, Xiang S, Williams K, Bai W, et al Ubiquitin-specific Peptidase 10 (USP10) Deubiquitinates and Stabilizes MutS Homolog 2 (MSH2) to Regulate Cellular Sensitivity to DNA Damage. The Journal of biological chemistry. 2016;291(20):10783–91. 10.1074/jbc.M115.700047 26975374 PMC4865924

[49] O'Hagan RC, Ohh M, David G, de Alboran IM, Alt FW, Kaelin WG Jr., et al Myc-enhanced expression of Cul1 promotes ubiquitin-dependent proteolysis and cell cycle progression. Genes & development. 2000;14(17):2185–91. 10970882 10.1101/gad.827200 PMC316894

[50] Nakayama K, Nagahama H, Minamishima YA, Matsumoto M, Nakamichi I, Kitagawa K, et al Targeted disruption of Skp2 results in accumulation of cyclin E and p27(Kip1), polyploidy and centrosome overduplication. The EMBO journal. 2000;19(9):2069–81. 10.1093/emboj/19.9.2069 10790373 PMC305685

[51] Holzer M, Krahling V, Amman F, Barth E, Bernhart SH, Carmelo VA, et al Differential transcriptional responses to Ebola and Marburg virus infection in bat and human cells. Scientific reports. 2016;6:34589 10.1038/srep34589 27713552 PMC5054393

[52] Dufner A, Kisser A, Niendorf S, Basters A, Reissig S, Schonle A, et al The ubiquitin-specific protease USP8 is critical for the development and homeostasis of T cells. Nature immunology. 2015;16(9):950–60. 10.1038/ni.3230 . 26214742

[53] MacDonald E, Urbe S, Clague MJ. USP8 controls the trafficking and sorting of lysosomal enzymes. Traffic. 2014;15(8):879–88. 10.1111/tra.12180 . 24894536

[54] Sevin M, Girodon F, Garrido C, de Thonel A. HSP90 and HSP70: Implication in Inflammation Processes and Therapeutic Approaches for Myeloproliferative Neoplasms. Mediators of inflammation. 2015;2015:970242 10.1155/2015/970242 26549943 PMC4624912

[55] Evans SS, Repasky EA, Fisher DT. Fever and the thermal regulation of immunity: the immune system feels the heat. Nature reviews Immunology. 2015;15(6):335–49. 10.1038/nri3843 25976513 PMC4786079

[56] Schroder M. Endoplasmic reticulum stress responses. Cellular and molecular life sciences: CMLS. 2008;65(6):862–94. 10.1007/s00018-007-7383-5 . 18038217 PMC11131897

[57] Lancini C, van den Berk PC, Vissers JH, Gargiulo G, Song JY, Hulsman D, et al Tight regulation of ubiquitin-mediated DNA damage response by USP3 preserves the functional integrity of hematopoietic stem cells. The Journal of experimental medicine. 2014;211(9):1759–77. 10.1084/jem.20131436 25113974 PMC4144738

[58] Chitta K, Paulus A, Akhtar S, Blake MK, Caulfield TR, Novak AJ, et al Targeted inhibition of the deubiquitinating enzymes, USP14 and UCHL5, induces proteotoxic stress and apoptosis in Waldenstrom macroglobulinaemia tumour cells. British journal of haematology. 2015;169(3):377–90. 10.1111/bjh.13304 25691154 PMC4846423

[59] Mosbech A, Lukas C, Bekker-Jensen S, Mailand N. The deubiquitylating enzyme USP44 counteracts the DNA double-strand break response mediated by the RNF8 and RNF168 ubiquitin ligases. The Journal of biological chemistry. 2013;288(23):16579–87. 10.1074/jbc.M113.459917 23615962 PMC3675593

[60] Grizenkova J, Akhtar S, Hummerich H, Tomlinson A, Asante EA, Wenborn A, et al Overexpression of the Hspa13 (Stch) gene reduces prion disease incubation time in mice. Proceedings of the National Academy of Sciences of the United States of America. 2012;109(34):13722–7. 10.1073/pnas.1208917109 22869728 PMC3427081

[61] Ogrunc M, Martinez-Zamudio RI, Sadoun PB, Dore G, Schwerer H, Pasero P, et al USP1 Regulates Cellular Senescence by Controlling Genomic Integrity. Cell reports. 2016;15(7):1401–11. 10.1016/j.celrep.2016.04.033 . 27160904

[62] Popova A, Kzhyshkowska J, Nurgazieva D, Goerdt S, Gratchev A. Smurf2 regulates IL17RB by proteasomal degradation of its novel binding partner DAZAP2. Immunobiology. 2012;217(3):321–8. 10.1016/j.imbio.2011.10.004 . 22070932

[63] Liu YC, Penninger J, Karin M. Immunity by ubiquitylation: a reversible process of modification. Nature reviews Immunology. 2005;5(12):941–52. 10.1038/nri1731 . 16322747 PMC7096784

[64] Sandoval D, Hill S, Ziemba A, Lewis S, Kuhlman B, Kleiger G. Ubiquitin-conjugating enzyme Cdc34 and ubiquitin ligase Skp1-cullin-F-box ligase (SCF) interact through multiple conformations. The Journal of biological chemistry. 2015;290(2):1106–18. 10.1074/jbc.M114.615559 25425648 PMC4294478

[65] Jamart C, Gomes AV, Dewey S, Deldicque L, Raymackers JM, Francaux M. Regulation of ubiquitin-proteasome and autophagy pathways after acute LPS and epoxomicin administration in mice. BMC musculoskeletal disorders. 2014;15:166 10.1186/1471-2474-15-166 24885455 PMC4041039

[66] Lanneau D, Brunet M, Frisan E, Solary E, Fontenay M, Garrido C. Heat shock proteins: essential proteins for apoptosis regulation. Journal of cellular and molecular medicine. 2008;12(3):743–61. 10.1111/j.1582-4934.2008.00273.x 18266962 PMC4401125

[67] Gusev NB, Bukach OV, Marston SB. Structure, properties, and probable physiological role of small heat shock protein with molecular mass 20 kD (Hsp20, HspB6). Biochemistry Biokhimiia. 2005;70(6):629–37. . 16038604 10.1007/s10541-005-0162-8

[68] Haslbeck M, Vierling E. A first line of stress defense: small heat shock proteins and their function in protein homeostasis. Journal of molecular biology. 2015;427(7):1537–48. 10.1016/j.jmb.2015.02.002 25681016 PMC4360138

[69] Chitra P, Bakthavatsalam B, Palvannan T. Beta-2 microglobulin as an immunological marker to assess the progression of human immunodeficiency virus infected patients on highly active antiretroviral therapy. Clinica chimica acta; international journal of clinical chemistry. 2011;412(11–12):1151–4. 10.1016/j.cca.2011.01.037 . 21300045

[70] Xie J, Wang Y, Freeman ME 3rd, Barlogie B, Yi Q. Beta 2-microglobulin as a negative regulator of the immune system: high concentrations of the protein inhibit in vitro generation of functional dendritic cells. Blood. 2003;101(10):4005–12. 10.1182/blood-2002-11-3368 . 12531797

[71] Cui Z, Wang P, Sun L, Liu H, Yang J, Li X, et al Lipopolysaccharide-evoked HSPA12B expression by activation of MAPK cascade in microglial cells of the spinal cord. Journal of the neurological sciences. 2010;294(1–2):29–37. 10.1016/j.jns.2010.04.009 . 20488464

[72] Kang L, Zhang G, Yan Y, Ke K, Wu X, Gao Y, et al The role of HSPA12B in regulating neuronal apoptosis. Neurochemical research. 2013;38(2):311–20. 10.1007/s11064-012-0922-y . 23292195

[73] Fullgrabe J, Hajji N, Joseph B. Cracking the death code: apoptosis-related histone modifications. Cell death and differentiation. 2010;17(8):1238–43. 10.1038/cdd.2010.58 . 20467440

[74] Dumoux M, Menny A, Delacour D, Hayward RD. A Chlamydia effector recruits CEP170 to reprogram host microtubule organization. Journal of cell science. 2015;128(18):3420–34. 10.1242/jcs.169318 26220855 PMC4582400

[75] Banerjee S, de Freitas A, Friggeri A, Zmijewski JW, Liu G, Abraham E. Intracellular HMGB1 negatively regulates efferocytosis. Journal of immunology. 2011;187(9):4686–94. 10.4049/jimmunol.1101500 21957148 PMC3197890

[76] Tao T, Shi Y, Han D, Luan W, Qian J, Zhang J, et al TPM3, a strong prognosis predictor, is involved in malignant progression through MMP family members and EMT-like activators in gliomas. Tumour biology: the journal of the International Society for Oncodevelopmental Biology and Medicine. 2014;35(9):9053–9. 10.1007/s13277-014-1974-1 . 24913705

[77] Cho KI, Patil H, Senda E, Wang J, Yi H, Qiu S, et al Differential loss of prolyl isomerase or chaperone activity of Ran-binding protein 2 (Ranbp2) unveils distinct physiological roles of its cyclophilin domain in proteostasis. The Journal of biological chemistry. 2014;289(8):4600–25. 10.1074/jbc.M113.538215 24403063 PMC3931022

[78] Zuehlke AD, Beebe K, Neckers L, Prince T. Regulation and function of the human HSP90AA1 gene. Gene. 2015;570(1):8–16. 10.1016/j.gene.2015.06.018 26071189 PMC4519370

[79] Zhang Z, Lin W, Li X, Cao H, Wang Y, Zheng SJ. Critical role of eukaryotic elongation factor 1 alpha 1 (EEF1A1) in avian reovirus sigma-C-induced apoptosis and inhibition of viral growth. Archives of virology. 2015;160(6):1449–61. 10.1007/s00705-015-2403-5 . 25854689

[80] Araki N. Role of microtubules and myosins in Fc gamma receptor-mediated phagocytosis. Frontiers in bioscience: a journal and virtual library. 2006;11:1479–90. . 16368530 10.2741/1897

[81] Ritho J, Arold ST, Yeh ET. A Critical SUMO1 Modification of LKB1 Regulates AMPK Activity during Energy Stress. Cell reports. 2015;12(5):734–42. 10.1016/j.celrep.2015.07.002 . 26212320

[82] Singla V, Romaguera-Ros M, Garcia-Verdugo JM, Reiter JF. Ofd1, a human disease gene, regulates the length and distal structure of centrioles. Developmental cell. 2010;18(3):410–24. 10.1016/j.devcel.2009.12.022 20230748 PMC2841064

[83] Liu J, Xu D, Wang Q, Zheng D, Jiang X, Xu L. LPS induced miR-181a promotes pancreatic cancer cell migration via targeting PTEN and MAP2K4. Digestive diseases and sciences. 2014;59(7):1452–60. 10.1007/s10620-014-3049-y . 24532253

[84] Leduc F, Maquennehan V, Nkoma GB, Boissonneault G. DNA damage response during chromatin remodeling in elongating spermatids of mice. Biology of reproduction. 2008;78(2):324–32. 10.1095/biolreprod.107.064162 . 18032420

[85] Geoffroy MC, Hay RT. An additional role for SUMO in ubiquitin-mediated proteolysis. Nature reviews Molecular cell biology. 2009;10(8):564–8. 10.1038/nrm2707 . 19474794

[86] Kyriakis JM, Avruch J. Mammalian MAPK signal transduction pathways activated by stress and inflammation: a 10-year update. Physiological reviews. 2012;92(2):689–737. 10.1152/physrev.00028.2011 . 22535895

[87] Ombrello MJ, Remmers EF, Sun G, Freeman AF, Datta S, Torabi-Parizi P, et al Cold urticaria, immunodeficiency, and autoimmunity related to PLCG2 deletions. The New England journal of medicine. 2012;366(4):330–8. 10.1056/NEJMoa1102140 22236196 PMC3298368

[88] Wang W, Jiang Q, Argentini M, Cornu D, Gigant B, Knossow M, et al Kif2C minimal functional domain has unusual nucleotide binding properties that are adapted to microtubule depolymerization. The Journal of biological chemistry. 2012;287(18):15143–53. 10.1074/jbc.M111.317859 22403406 PMC3340219

[89] Naro C, Barbagallo F, Chieffi P, Bourgeois CF, Paronetto MP, Sette C. The centrosomal kinase NEK2 is a novel splicing factor kinase involved in cell survival. Nucleic acids research. 2014;42(5):3218–27. 10.1093/nar/gkt1307 24369428 PMC3950702

[90] Subramanian R, Wilson-Kubalek EM, Arthur CP, Bick MJ, Campbell EA, Darst SA, et al Insights into antiparallel microtubule crosslinking by PRC1, a conserved nonmotor microtubule binding protein. Cell. 2010;142(3):433–43. 10.1016/j.cell.2010.07.012 20691902 PMC2966277

[91] Fourest-Lieuvin A, Peris L, Gache V, Garcia-Saez I, Juillan-Binard C, Lantez V, et al Microtubule regulation in mitosis: tubulin phosphorylation by the cyclin-dependent kinase Cdk1. Molecular biology of the cell. 2006;17(3):1041–50. 10.1091/mbc.E05-07-0621 16371510 PMC1382296

[92] Hamilton DH, Huang B, Fernando RI, Tsang KY, Palena C. WEE1 inhibition alleviates resistance to immune attack of tumor cells undergoing epithelial-mesenchymal transition. Cancer research. 2014;74(9):2510–9. 10.1158/0008-5472.CAN-13-1894 24626094 PMC4011139

[93] Glatigny S, Wagner CA, Bettelli E. Cutting Edge: Integrin alpha4 Is Required for Regulatory B Cell Control of Experimental Autoimmune Encephalomyelitis. Journal of immunology. 2016;196(9):3542–6. 10.4049/jimmunol.1502614 27016608 PMC4868662

[94] Li C, Jensen VL, Park K, Kennedy J, Garcia-Gonzalo FR, Romani M, et al MKS5 and CEP290 Dependent Assembly Pathway of the Ciliary Transition Zone. PLoS biology. 2016;14(3):e1002416 10.1371/journal.pbio.1002416 26982032 PMC4794247

[95] Lee SY, Ramirez J, Franco M, Lectez B, Gonzalez M, Barrio R, et al Ube3a, the E3 ubiquitin ligase causing Angelman syndrome and linked to autism, regulates protein homeostasis through the proteasomal shuttle Rpn10. Cellular and molecular life sciences: CMLS. 2014;71(14):2747–58. 10.1007/s00018-013-1526-7 . 24292889 PMC11113982

[96] Lord CJ, Ashworth A. The DNA damage response and cancer therapy. Nature. 2012;481(7381):287–94. 10.1038/nature10760 . 22258607

[97] Halpain S, Dehmelt L. The MAP1 family of microtubule-associated proteins. Genome biology. 2006;7(6):224 10.1186/gb-2006-7-6-224 16938900 PMC1779536

[98] Ikeda T. Parkin-co-regulated gene (PACRG) product interacts with tubulin and microtubules. FEBS letters. 2008;582(10):1413–8. 10.1016/j.febslet.2008.02.081 . 18387367

[99] Byron A, Askari JA, Humphries JD, Jacquemet G, Koper EJ, Warwood S, et al A proteomic approach reveals integrin activation state-dependent control of microtubule cortical targeting. Nature communications. 2015;6:6135 10.1038/ncomms7135 25609142 PMC4317495

[100] Merriam EB, Millette M, Lumbard DC, Saengsawang W, Fothergill T, Hu X, et al Synaptic regulation of microtubule dynamics in dendritic spines by calcium, F-actin, and drebrin. The Journal of neuroscience: the official journal of the Society for Neuroscience. 2013;33(42):16471–82. 10.1523/JNEUROSCI.0661-13.2013 24133252 PMC3797370

[101] He F, Chen H, Probst-Kepper M, Geffers R, Eifes S, Del Sol A, et al PLAU inferred from a correlation network is critical for suppressor function of regulatory T cells. Molecular systems biology. 2012;8:624 10.1038/msb.2012.56 23169000 PMC3531908

[102] Kuo JC. Mechanotransduction at focal adhesions: integrating cytoskeletal mechanics in migrating cells. Journal of cellular and molecular medicine. 2013;17(6):704–12. 10.1111/jcmm.12054 23551528 PMC3823174

[103] Vanamala J, Reddivari L, Radhakrishnan S, Tarver C. Resveratrol suppresses IGF-1 induced human colon cancer cell proliferation and elevates apoptosis via suppression of IGF-1R/Wnt and activation of p53 signaling pathways. BMC cancer. 2010;10:238 10.1186/1471-2407-10-238 20504360 PMC2891636

[104] Mangahas PM, Yu X, Miller KG, Zhou Z. The small GTPase Rab2 functions in the removal of apoptotic cells in Caenorhabditis elegans. The Journal of cell biology. 2008;180(2):357–73. 10.1083/jcb.200708130 18227280 PMC2213587

[105] Neely KM, Green KN, LaFerla FM. Presenilin is necessary for efficient proteolysis through the autophagy-lysosome system in a gamma-secretase-independent manner. The Journal of neuroscience: the official journal of the Society for Neuroscience. 2011;31(8):2781–91. 10.1523/JNEUROSCI.5156-10.2010 21414900 PMC3064964

[106] Kawabe J, Okumura S, Nathanson MA, Hasebe N, Ishikawa Y. Caveolin regulates microtubule polymerization in the vascular smooth muscle cells. Biochemical and biophysical research communications. 2006;342(1):164–9. 10.1016/j.bbrc.2006.01.125 . 16480946

