# Supplementary figures and images for: The responses of lungs and adjacent lymph nodes in responding to Yersinia pestis infection: A transcriptomic study using a non-human primate model
Source: PLoS One. 2019 Feb 21;14(2):e0209592. doi: 10.1371/journal.pone.0209592 (PMC6383991; doi:10.1371/journal.pone.0209592)

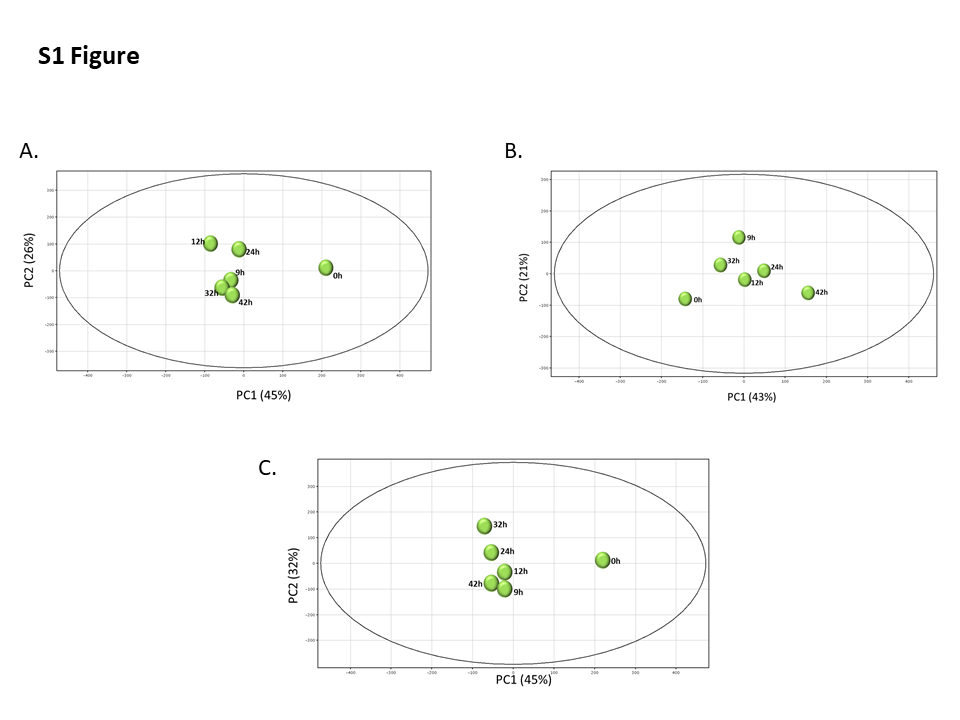

Supplement: S1 Fig — Each circle is labeled by the corresponding time points. (A) Submandibular LN. (B) Lungs. (C) Mediastinal LN. (TIF) [file pone.0209592.s001.TIF]

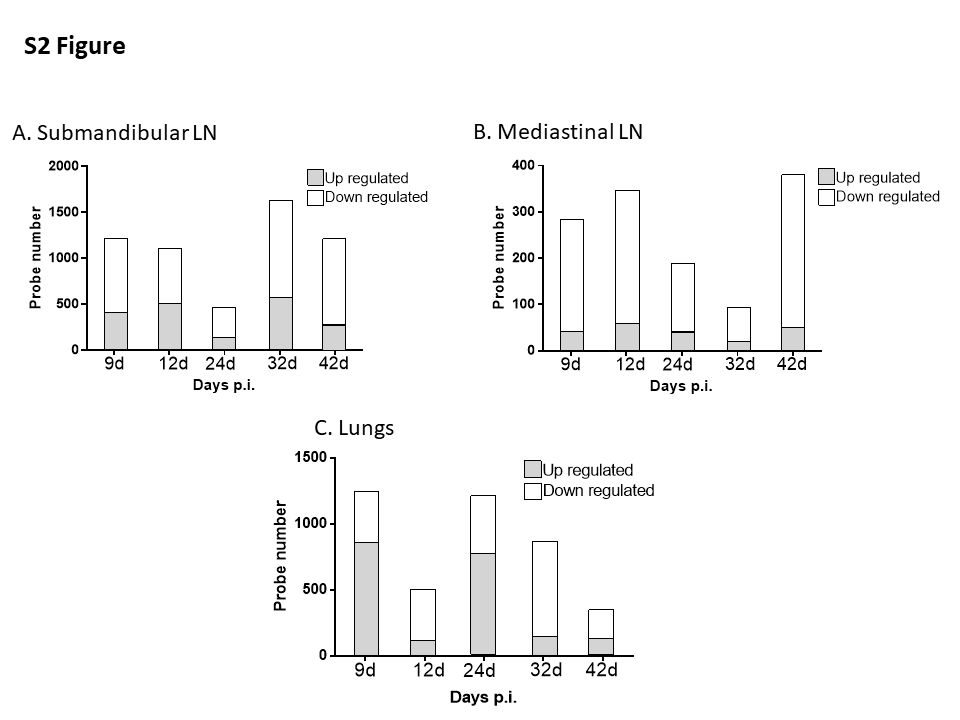

Supplement: S2 Fig — (A) submandibular lymph node, (B) mediastinal lymph node and (C) lungs. The shaded bar represents up regulated and clear bar represents down regulated probes. (TIF) [file pone.0209592.s002.TIF]

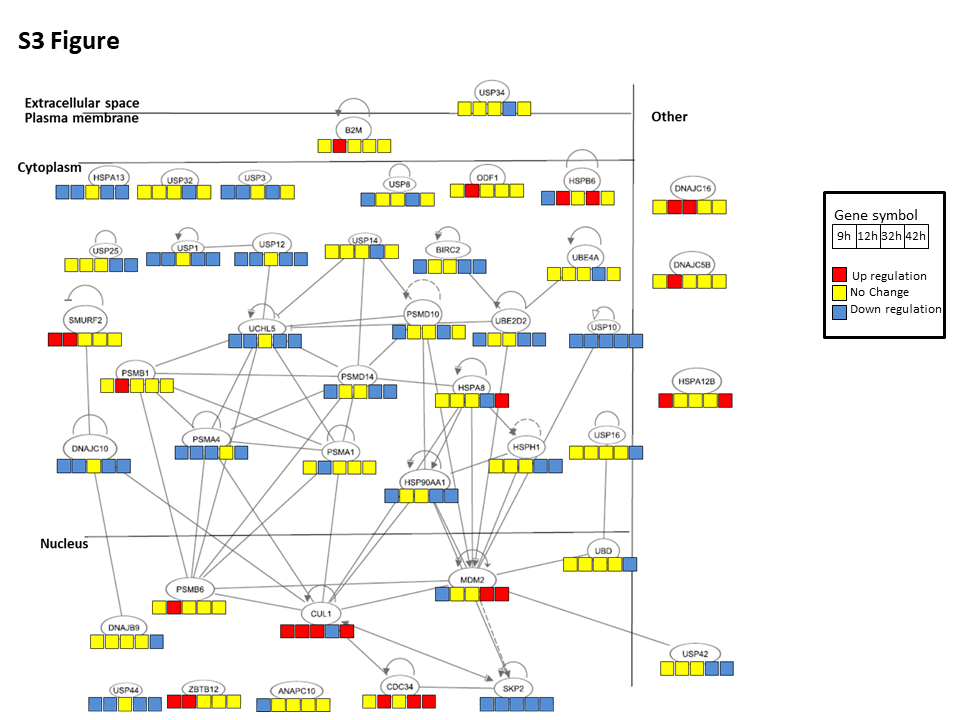

Supplement: S3 Fig — The diagram depicts the cellular locations of the proteins encoded by the genes enriching the ubiquitin network. The temporal expression of individual genes are shown under each gene symbol. To note, there was no ubiquitin-associated gene significantly altered at 24 h p.i.; hence gene expression at 9 h, 12 h, 32 h and 42 h are shown. (TIF) [file pone.0209592.s003.TIF]

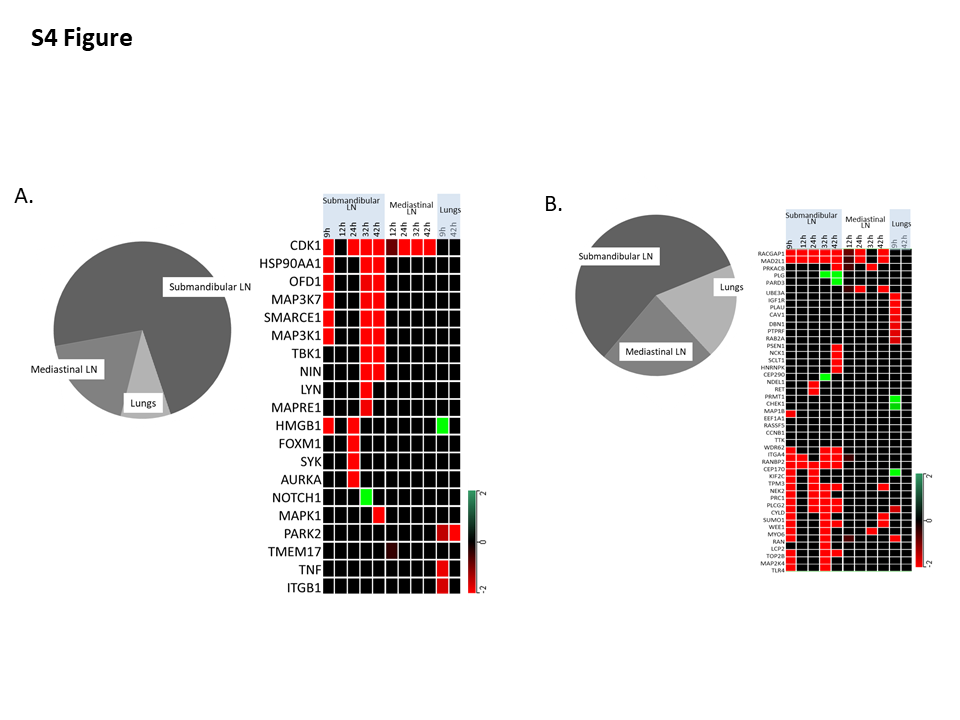

Supplement: S4 Fig — (A) Primary tier. (B) Secondary tier. The pie chart shows the distribution of genes across three organs, and the corresponding hierarchical matrix shows the temporal pattern of transcriptional expression of these genes in three organs. A scale depicting the range of color corresponding to the gene expression levels is shown at right. Each row corresponds to one particular gene. LN: Lymph node. (TIF) [file pone.0209592.s004.TIF]

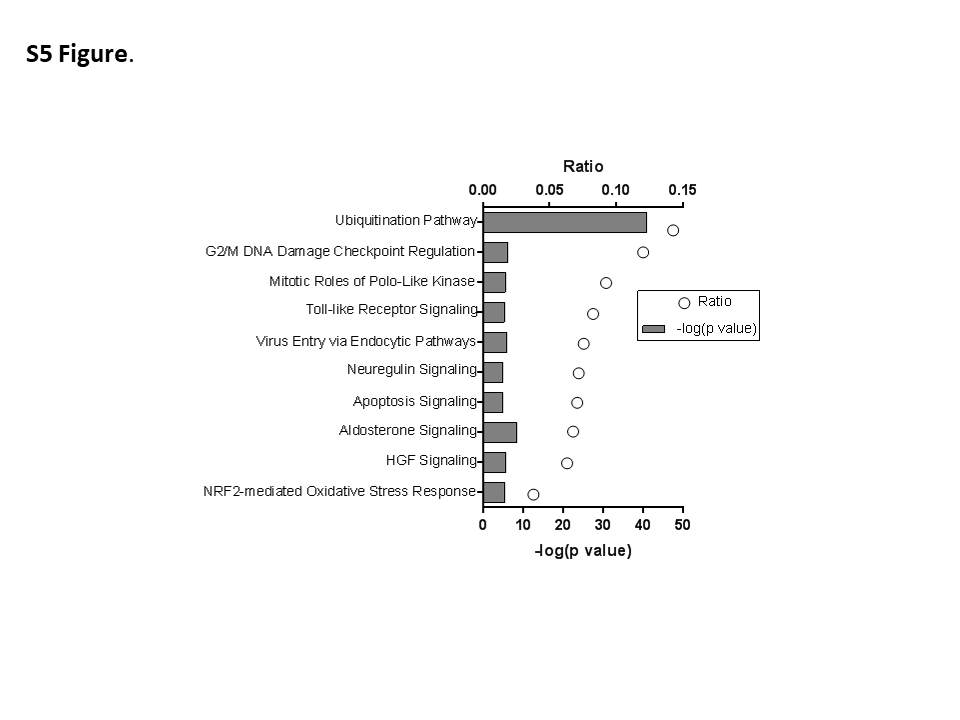

Supplement: S5 Fig — The top axis represents the ratio of the genes altered by Y. pestis infection and the entire gene list of this canonical network. The bottom axis represents–log(p value), where the p value represents the enrichment factor calculated by the hypergeometric test. (TIF) [file pone.0209592.s005.TIF]

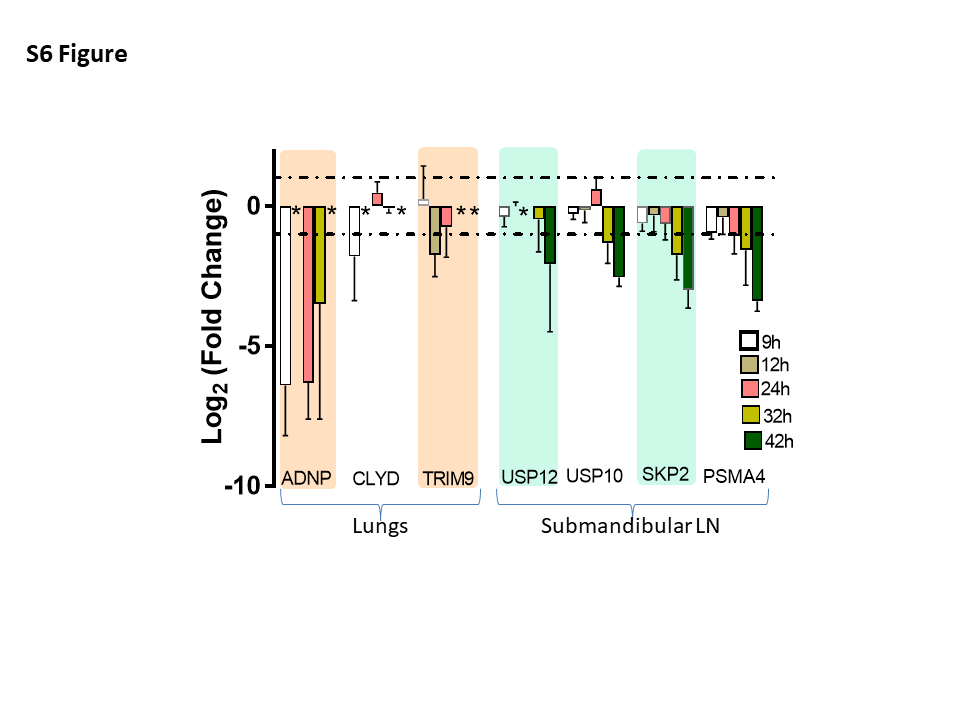

Supplement: S6 Fig — For each gene, 5 time points were represented with error bars. The assay results showing zero fold changes were marked as *. (TIF) [file pone.0209592.s006.TIF]
